# A light-controlled multi-step drug release nanosystem targeting tumor hypoxia for synergistic cancer therapy†

**DOI:** 10.1039/d1sc01888d

**Published:** 2021-08-03

**Authors:** Bin Zhang, Zichen Xu, Wen Zhou, Zhikun Liu, Jian Zhao, Shaohua Gou

**Affiliations:** Jiangsu Province Hi-Tech Key Laboratory for Biomedical Research, Southeast University Nanjing 211189 China zhaojianzhaokuan@163.com 2219265800@qq.com; Pharmaceutical Research Center and School of Chemistry and Chemical Engineering, Southeast University Nanjing 211189 China; Nanjing Junruo Institute of Biomedicine Nanjing 211100 China

## Abstract

Hypoxia is a major obstacle for cancer therapy due to its association with cell proliferation, tumor distant metastasis, and treatment resistance. In this study, a hypoxia-activated bifunctional prodrug (CC5) was designed, synthesized and encapsulated by a photo-responsive ruthenium complex-derived polymer to yield a light-controlled multi-step drug release system (CC5-RuCa) for synergistic therapy against tumor hypoxia. Under NIR irradiation, CC5-RuCa not only generated ROS to kill the cancer cells in the exterior of the tumor but also released the prodrug CC5 with enhanced intratumoral penetration in the severe hypoxia region inside the tumor tissue. *In vivo* studies on MDA-MB-231 xenograft models revealed that CC5-RuCa with preferential accumulation in the tumor exhibited highly efficient tumor regression through the synergistic effect of photodynamic therapy and hypoxia-activated chemotherapy.

## Abstract 2

A photo-responsive ruthenium complex-derived micelle (CC5-RuCa) loaded with CC5 was prepared for light and tumor microenvironment-controlled multistage drug-release.

## Graphical abstract

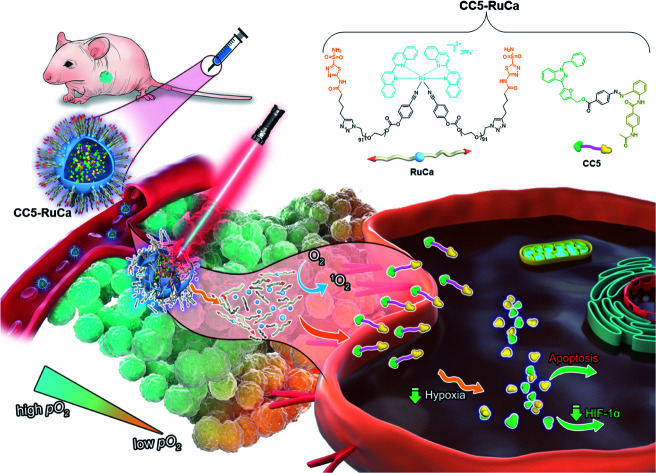

## Introduction

Hypoxia, a hallmark of most solid tumors, is one of the major obstacles for cancer therapy owing to its multiple contributions to the compromised therapeutic effects and resistance to anticancer therapies including radiation therapy (RT), chemotherapy, photodynamic therapy (PDT) and sonodynamic therapy (SDT).^1–6^ In addition, hypoxia has been found to be highly correlated with tumor invasiveness, metastasis and recurrence, and is therefore identified as a negative prognostic indicator for cancer therapy.^7–9^ In view of the adverse effects of hypoxia, targeting tumor hypoxia is an appealing strategy to achieve an effective cancer treatment.

To date, various approaches have been developed to fight against tumor hypoxia according to the unique characteristics of tumor hypoxic microenvironments.^10–12^ One of the effective strategies is to design hypoxia-activated prodrugs, which has shown great potential in clinical application. For example, several hypoxia-activated prodrugs, such as tirapazamine (TPZ), PR-104 and TH-302, are under preclinical and clinical evaluation, which could be selectively activated to cytotoxic agents by reductase at hypoxia, thus converting tumor hypoxia from an impediment to a therapy advantage.^13–18^ However, through single hypoxia-activated chemotherapy it is hard to achieve satisfactory results in clinical trials.^19^ Consequently, the development of a synergistic strategy by combining a hypoxia-activated target drug with other hypoxia-targeted inhibitors in hypoxia-dependent processes in a small-molecule entity is valuable and meaningful.

**Fig. 1:**
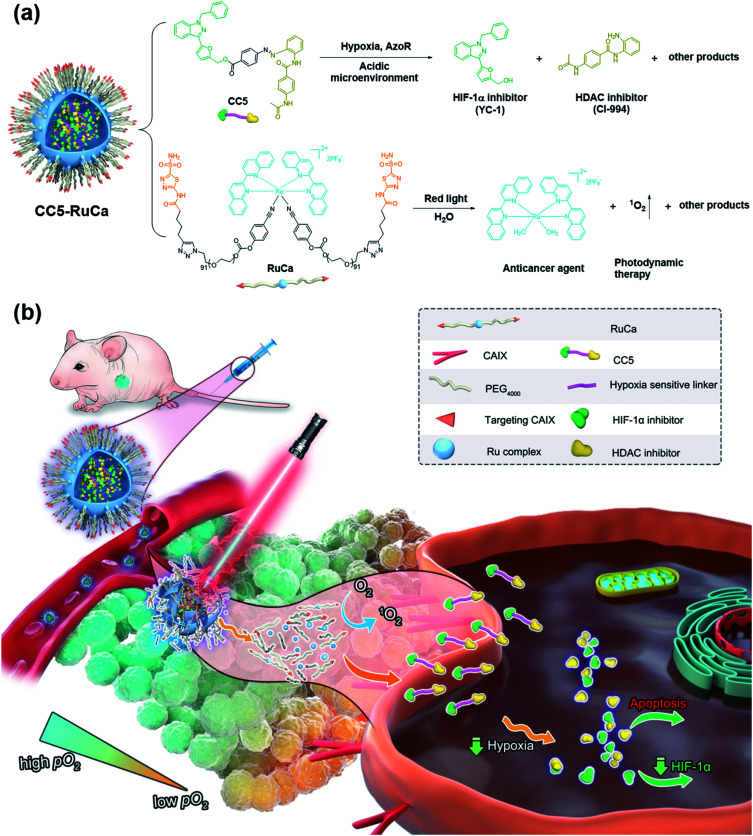
(a) Chemical structures of the prodrug CC5 and the carrier RuCa, and their release processes. (b) Schematic illustration of extracellular and intracellular multi-step drug release of CC5-RuCa for hypoxic tumor therapy.

Hypoxia inducible factor-1α (HIF-1α), a transcription factor that responds to a hypoxic environment, was found overexpressed in more than 90% of colon, lung, and prostate cancers. As associated with tumor progression, metastasis and resistance to treatment, HIF-1α inhibitors have shown great promise in cancer therapy.^20–23^ 1-Benzyl-3-(5′-hydroxymethyl-2′-furyl)indazole (YC-1), an effective HIF-1α inhibitor, can inhibit the overexpression of HIF-1α induced by hypoxia, thus exerting a potent antitumor effect.^24,25^ Based on the above, we attempted to develop a bifunctional prodrug, which contains both a hypoxia-activated prodrug and a hypoxia-targeted inhibitor (YC-1) in a small-molecule entity. So a hypoxia-activated self-immolative prodrug (CC5), composed of an anticancer agent (CI-944, a histone deacetylase inhibitor)^26^ and YC-1 *via* a hypoxia-responsive azobenzene linker, was firstly designed and synthesized. Under hypoxia, CC5 would undergo self-immolative cleavage to release CI-944 and YC-1 for synergistic therapy due to the reduction of the azo bond by reductive species (Fig. 1). However, the therapeutic efficacy of CC5 would not be successfully achieved due to the following limitations as predicted. One is the high oxygen concentration in the exterior of the tumor, which will doubtlessly decrease the sensitivity of the hypoxia-responsive prodrug and compromise its therapeutic efficacy.^9,27^ The other is the poor performance of the small molecule prodrug in biodistribution and pharmacokinetics because of its short blood circulation time, thereby resulting in insufficient drug accumulation in tumors.^28,29^ Therefore, an efficient strategy to overcome these limitations is necessary.

PDT is a promising noninvasive cancer therapy modality, which can convert endogenous molecular oxygen into toxic reactive oxygen species (ROS) upon irradiation and further lead to cell death.^30–33^ In our system, the consumption of oxygen would exacerbate the tumor hypoxia, which could improve the sensitivity of CC5 and promote the release of CI-944 and YC-1. Additionally, it is known that the photolabile ruthenium complex-derived polymeric micelles can not only generate ROS under irradiation, but also result in the ligand dissociation, enabling the drug burst release and concomitant PDT.^34–37^ Thus, it is rational to design photolabile ruthenium complex-derived polymeric micelles as a drug delivery system for overcoming the limitations of CC5. Herein, we developed a light-controlled ruthenium complex-derived drug release nanosystem (CC5-RuCa) with deep tumor penetration ability to synergize chemotherapeutic and photodynamic treatments against hypoxia in tumor (Fig. 1). Notably, our system has several attractive features as follows. (i) CC5-RuCa containing a photo-responsive ruthenium polymer as the drug loading carrier can self-assemble into nanoparticles (NPs), offering advantages in promoting its blood circulation time and accumulation in the tumor through the EPR effect. (ii) Since carbonic anhydrase IX (CAIX) is widely expressed on the surface of hypoxic tumor cells,^38,39^ acetazolamide (an inhibitor for CAIX) was covalently conjugated at the end of the ruthenium polymer to further improve its specificity and selectivity. (iii) The photo-responsive ruthenium complex-derived polymer can not only efficiently release the Ru(ii) anticancer agent and CC5 upon NIR light irradiation, but also act as a photosensitizer to kill cancer cells in the exterior of the tumor. (iv) The released small molecule prodrug CC5 has the potential to overcome the limitation of the poor penetration efficiency of the nanoparticles (100–150 nm) due to their large diffusion barrier in the tumor space, enabling an enhanced penetration and intratumoral permeability. (v) CC5 with increased tumor penetration can be sensitively reduced by azoreductase to release cytotoxic CI-944 and YC-1 in the severe hypoxia region inside the tumor tissue (oxygen concentration < 3%). Compared with the small molecule prodrug CC5, the derived formulation (*i.e.* CC5-RuCa) presented better biocompatibility, pharmacokinetic behavior and higher tumor selectivity, demonstrating enhanced anticancer activity against MDA-MB-231 cancer.

## Results

### Synthesis and hypoxia response of CC5

CC5 was prepared from 4-aminobenzoic acid *via* a five-step route by oxidation, diazotization, hydrolysis and esterification reactions (Scheme S1, ESI†), and its structure was characterized by ^1^H and ^13^C NMR spectroscopy along with electrospray ionization mass spectrometry (Fig. S1–S10, ESI†).

**Fig. 2:**
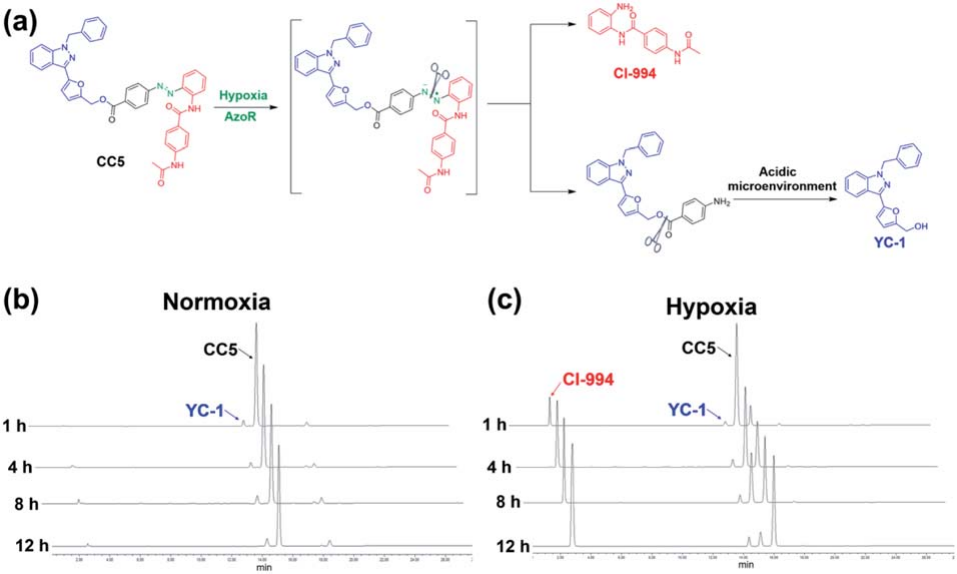
(a) The cleavage pathway of CC5 under hypoxic and acidic conditions. HPLC analysis of CC5 under (b) normoxic and (c) hypoxic microenvironments at pH 7.4.

Azo-reductase, as an important family of reductases, is highly expressed in hypoxic tumors and can reduce the azobenzene groups to anilines in a progressive pattern.^40–42^ To validate the hypoxia-induced reduction of CC5 *in vitro*, we applied the HPLC technique to evaluate the release of CI-994 and YC-1 from CC5 by co-incubation with rat liver microsomes and NADPH at pH 7.4 and pH 5.0, respectively.^42,43^ As shown in Fig. 2b and c, CC5 degraded rapidly under hypoxic conditions (1% O_2_), and generated a new peak with a retention time of 2.7 min, which was assigned to CI-994. In contrast, negligible degradation of CC5 was observed under normoxia, hinting that the azo bond of CC5 could be selectively cleaved under hypoxia. Besides, a small amount of YC-1 was observed both in hypoxia and normoxia after 12 h incubation. Significantly, the release of YC-1 was greatly accelerated at pH 5.0 (Fig. S11, ESI†), suggesting that the acidic microenvironment of the tumor tissue is beneficial for YC-1 release.

### *In vitro* antiproliferative activity of CC5

**Fig. 3:**
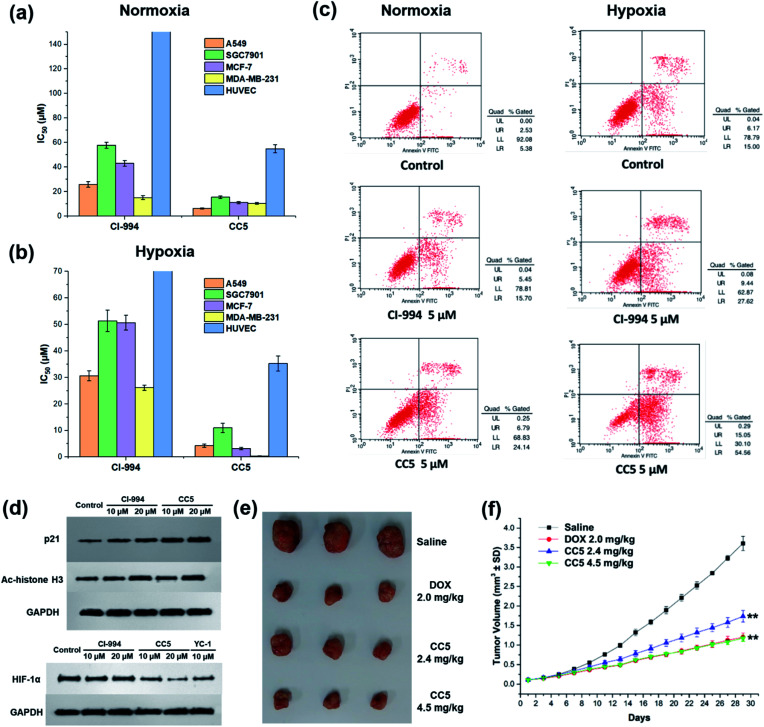
IC_50_ values of CI-994 and CC5 against different cell lines under (a) normoxic or (b) hypoxic conditions. (c) Flow cytometry analysis for apoptosis of MDA-MB-231 cells after 48 h treatment with CI-994 and CC5 under normoxic or hypoxic conditions. (d) Effects of CC5 and CI-994 on HIF-1α, p21 and Ac-histone H3 protein expression. (e) Gross solid tumor images of mice after treatment with saline, DOX, and CC5. (f) The tumor growth curves with different treatments. **p < 0.01 *versus* PBS (two-tailed Student's *t*-test). Error bars indicate standard deviation (SD; *n* = 3).

*In vitro* cytotoxic activities of CC5 against five cell lines including the normal human umbilical vein endothelial cells (HUVEC) have been evaluated with CI-994 and YC-1 as controls by MTT assay. As shown in Fig. 3a and Table S1 (ESI†), YC-1 displayed negligible cytotoxicity (>200 μM) against all the tested cancer cell lines under normoxia, while CI-994 and CC5 showed certain cytotoxicity. And interestingly, CC5 showed superior cytotoxicity to CI-994 under the same conditions. However, CC5 under hypoxia exhibited markedly improved cytotoxic activity toward the tested cancer cells, especially against MDA-MB-231 cells with an IC_50_ value of 0.34 μM (Fig. 3b and Table S2, ESI†). In contrast, the cytotoxicity of CI-994 against the tested cancer cells under hypoxia was comparable to that under normoxic conditions. It is noteworthy that CC5 had much lower cytotoxicity against HUVEC cells than against cancer cells under hypoxia. Overall, the significant cytotoxicity of CC5 confirmed its high potential as an anticancer agent, especially under hypoxia.

### CC5 induced apoptotic cell death and western blot analysis

The potential of CC5 and CI-994 to induce cell death was investigated by Annexin V-FITC/propidium iodide (PI) assay. The tested compounds were incubated with MDA-MB-231 cells for 48 h at a concentration of 5 μM. As shown in Fig. 3c, the apoptotic rates of MDA-MB-231 cells treated with CC5 (30.9%) and CI-994 (21.2%) increased as compared with that of the untreated cells (7.9%) under normoxic conditions. Significantly, once hypoxia was conducted, CC5 induced a 2.3-fold increased incidence of early to late stage apoptosis in MDA-MB-231 cells compared with that in normoxia. Additionally, the apoptotic rate of CC5 (69.6%) was much higher than that of CI-994 (37.1%), proving that CC5 had advantages over CI-994 to induce cell apoptosis under hypoxia. This study further confirmed the potent cytotoxicity of CC5 toward hypoxic MDA-MB-231 cells.

Histone deacetylase inhibitors (HDACIs), as an important class of antitumor drugs, were capable of either altering gene transcription or inhibiting HDAC activity, thereby affecting the cell growth inhibition, differentiation, apoptosis, and tumor angiogenesis.^44–46^ Previous studies have shown that HDACIs not only induce histone H3 hyperacetylation (Ac-histone H3), but also up-regulate the expression of the p21 gene.^47^ Thus, the expressions of Ac-histone H3 and p21 proteins in MDA-MB-231 cells were evaluated by western blot assay. As depicted in Fig. 3d, both CC5 and CI-994 can up-regulate the expression of Ac-histone H3 and p21 proteins. Besides, the inhibition of HIF-1α protein accumulation by CC5 was also investigated by western blot assay. As illustrated in Fig. 3d, CI-994 had no effect on HIF-1α accumulation, while CC5 and YC-1 could down-regulate the level of HIF-1α protein expression under hypoxic conditions.

### *In vivo* antitumor efficacy of CC5

The *in vivo* tumor therapeutic effect of CC5 on the MDA-MB-231 cell xenograft mice model together with doxorubicin (DOX) as a positive drug was studied. The mice were randomly divided into four groups: (1) saline; (2) DOX (2.0 mg kg^−1^); (3) CC5 (2.4 mg kg^−1^, equimolar dosage to DOX); (4) CC5 (4.5 mg kg^−1^). The administration of CC5 efficiently inhibited the growth of the MDA-MB-231 tumor, in a dose-dependent manner, with 59.3% and 70.8% reduction in tumor weight at concentrations of 2.4 and 4.5 mg kg^−1^, respectively (Fig. 3f and S12, ESI†). Significantly, the antitumor efficacy of CC5 (4.5 mg kg^−1^) was comparable to that of DOX (69.3% tumor-suppression-rates). However, body weights of the mice in both DOX and CC5 groups decreased (Fig. S13, ESI†), indicating the low biocompatibility and potent systemic toxicity of DOX and CC5 *in vivo*.

### Preparation and characterization of RuCa and CC5-RuCa NPs

**Fig. 4:**
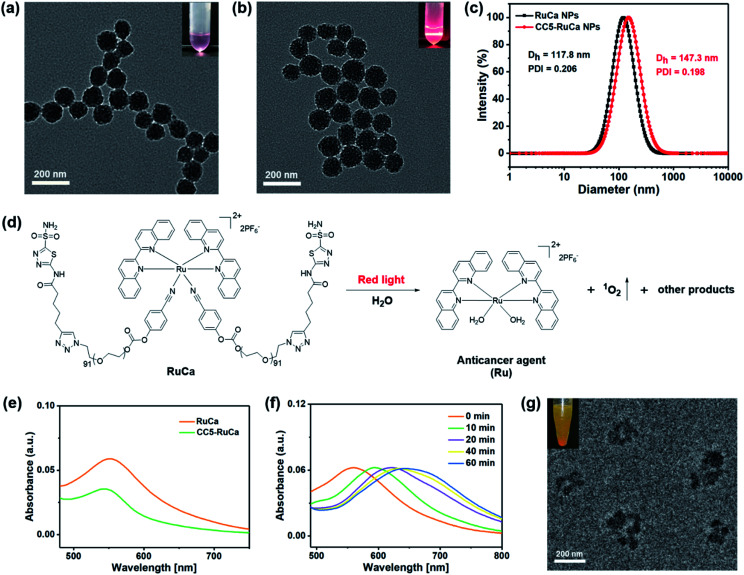
TEM images of (a) RuCa and (b) CC5-RuCa NPs. Scale bar: 200 nm. (c) Hydrodynamic diameter of RuCa and CC5-RuCa NPs. (d) Red light (660 nm, 30 mW cm^−2^) induced degradation of RuCa. (e) UV-vis spectra of RuCa and CC5-RuCa NPs in water. (f) UV-vis absorption spectra of RuCa NPs under 660 nm red light irradiation (30 mW cm^−2^) for different time periods. (g) TEM image of RuCa NPs after 10 min laser irradiation. Scale bar: 200 nm.

In the preparation of RuCa, we firstly synthesized the acetazolamide-containing polymer CA-PEG *via* multi-step routes (Scheme S2, ESI†). CA-PEG was then activated by reaction with 4-nitrobenzene chloroformate, and finally terminated by the Ru-containing monomer [Ru(Biq)_2_(Cya)_2_](PF_6_)_2_ (Biq = 2,2′-biquinoline, Cya = 4-cyanophenol). ^1^H NMR spectral data of RuCa confirmed the conjugation of [Ru(Biq)_2_(Cya)_2_](PF_6_)_2_ and CA-PEG (Fig. S19, ESI†). Self-assembled nanoparticles of RuCa and CC5-RuCa were prepared using a nanoprecipitation method according to the literature.^48–50^ The morphologies of RuCa and CC5-RuCa NPs were studied by transmission electron microscopy (TEM), which displayed uniform spherical shapes with average sizes of 92 nm and 115 nm, respectively (Fig. 4a and b). Dynamic light scattering (DLS) measurements revealed that the average hydrodynamic diameters of RuCa and CC5-RuCa were 118 nm and 147 nm with polydispersity indices (PDI) of 0.206 and 0.198, respectively (Fig. 4c). Besides, the drug-loading capacity and drug encapsulation efficiency of CC5 in CC5-RuCa were determined by HPLC, which were calculated to be about 14.5% and 72.5%, respectively (Fig. S21 and S22, ESI†).

### Photoresponse and singlet oxygen (^1^O_2_) generation of RuCa

The absorption spectra of RuCa and CC5-RuCa in water displayed broad metal-to-ligand charge transfer (MLCT) bands in 500–750 nm regions with an absorption maximum at around 553 nm (Fig. 4e), implying that RuCa and CC5-RuCa have the potential to be excited in the therapeutic window (600–900 nm). Therefore, the photolysis of RuCa was studied under irradiation with red light (660 nm, 30 mW cm^−2^) by using UV-Vis spectroscopy. As shown in Fig. 4f, the MLCT band of RuCa at 553 nm was red-shifted to around 650 nm, indicating that RuCa underwent ligand dissociation upon 660 nm irradiation. Besides, TEM images showed that the spherical structures of RuCa nanoparticles gradually ruptured with the increase of irradiation time, indicating that RuCa nanoparticles can undergo photodegradation upon laser irradiation (Fig. 4g and S23†). Moreover, precipitates were observed after 660 nm laser irradiation, confirming the photocleavage of RuCa nanoparticles.

^1^O_2_ generation capability of RuCa was investigated by using 9,10-anthracenediylbis(methylene)-dimalonic acid (ABDA) as a probe.^51,52^ As shown in Fig. S24 (ESI†), negligible changes of the absorption bands of ABDA were observed in the dark. Once 660 nm red light irradiation was conducted, the absorbance of ABDA was dramatically decreased, suggesting that RuCa is capable of producing ^1^O_2_ upon irradiation at 660 nm.

### *In vitro* cytotoxicity effects and ROS generation of RuCa and CC5-RuCa NPs

**Fig. 5:**
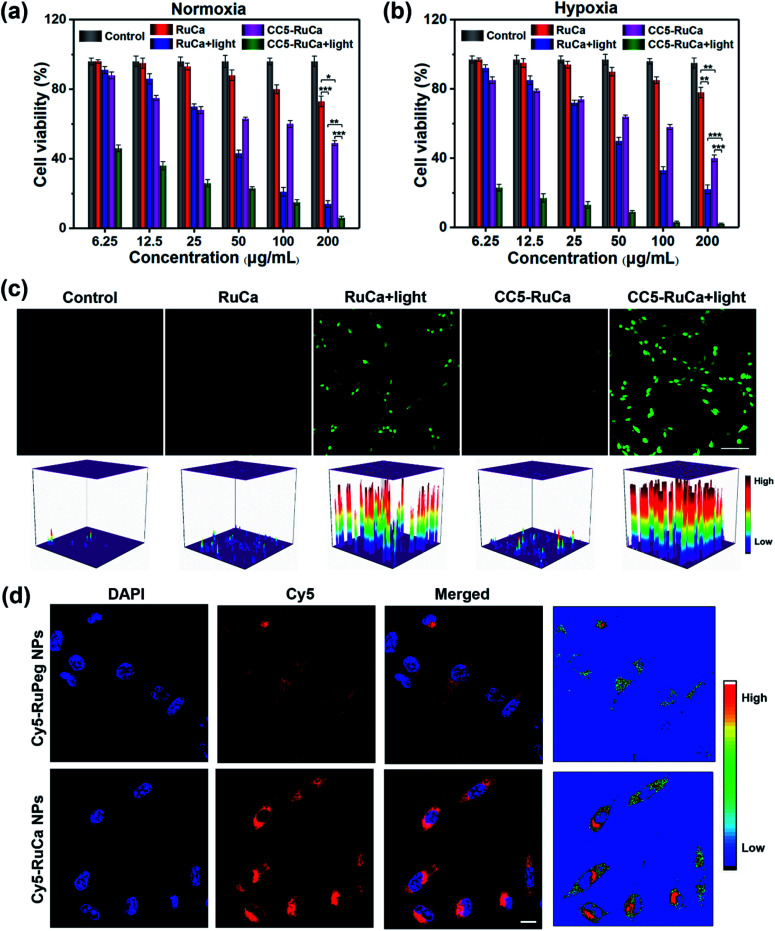
MDA-MB-231 cells treated with RuCa and CC5-RuCa NPs at different concentrations in the presence and absence of light irradiation (660 nm, 30 mW cm^−2^, 30 min) under (a) normoxic or (b) hypoxic conditions (*p < 0.05, **p < 0.01, ***p < 0.001). (c) CLSM images of ROS generation in MDA-MB-231 cells incubated with RuCa or CC5-RuCa NPs in the presence and absence of light irradiation. Scale bar: 80 μm. (d) CLSM images in MDA-MB-231 cells treated with Cy5-RuPeg or Cy5-RuCa NPs for 4 h. Scale bar: 20 μm.

The *in vitro* cytotoxicity of RuCa and CC5-RuCa NPs were evaluated against MDA-MB-231 cells. As shown in Fig. 5a and b, both RuCa and CC5-RuCa NPs exhibited negligible cytotoxicity in the dark even at high concentrations, but presented significant cytotoxicity upon exposure to 30 min laser irradiation. Particularly, CC5-RuCa NPs have an IC_50_ value of 0.81 μg mL^−1^ under hypoxia, which is much lower than that of RuCa NPs (68.04 μg mL^−1^) (Table S3, ESI†), suggesting the potential synergistic effect of PDT and hypoxia-activated chemotherapy.

The intracellular ROS generation in MDA-MB-231 cells induced by RuCa and CC5-RuCa NPs was evaluated with 2,7-dichlorodihydrofluorescein diacetate (DCFH-DA) as a ROS probe. As illustrated in Fig. 5c, no ROS production was observed for the MDA-MB-231 cells treated with RuCa and CC5-RuCa NPs in the absence of light irradiation. However, upon 660 nm laser irradiation, both RuCa and CC5-RuCa-treated MDA-MB-231 cells exhibited intense green fluorescence, demonstrating the potent intracellular ROS generation ability of RuCa and CC5-RuCa.

### *In vitro* cellular uptake and penetration behavior of Cy5-RuCa and Cy5-RuPeg NPs

Since carbonic anhydrase IX (CAIX) is overexpressed in MDA-MB-231 cells,^38,53^ the targeting ability of the vehicle was explored by observing the cellular uptake using confocal laser scanning microscopy (CLSM). RuCa loaded with fluorescent Cy5 dye (Cy5-RuCa NPs) was incubated with MDA-MB-231 cells for 6 h in the dark, together with Cy5-RuPeg NPs as a negative control. As shown in Fig. 5d, the intense red fluorescence in the cytoplasm was observed for Cy5-RuCa, indicative of the effective cellular accumulation in MDA-MB-231 cells. Meanwhile, the fluorescence intensity of Cy5-RuPeg was very weak, implying that the acetazolamide moiety plays a key role in mediating cellular uptake of RuCa in MDA-MB-231 cells.

**Fig. 6:**
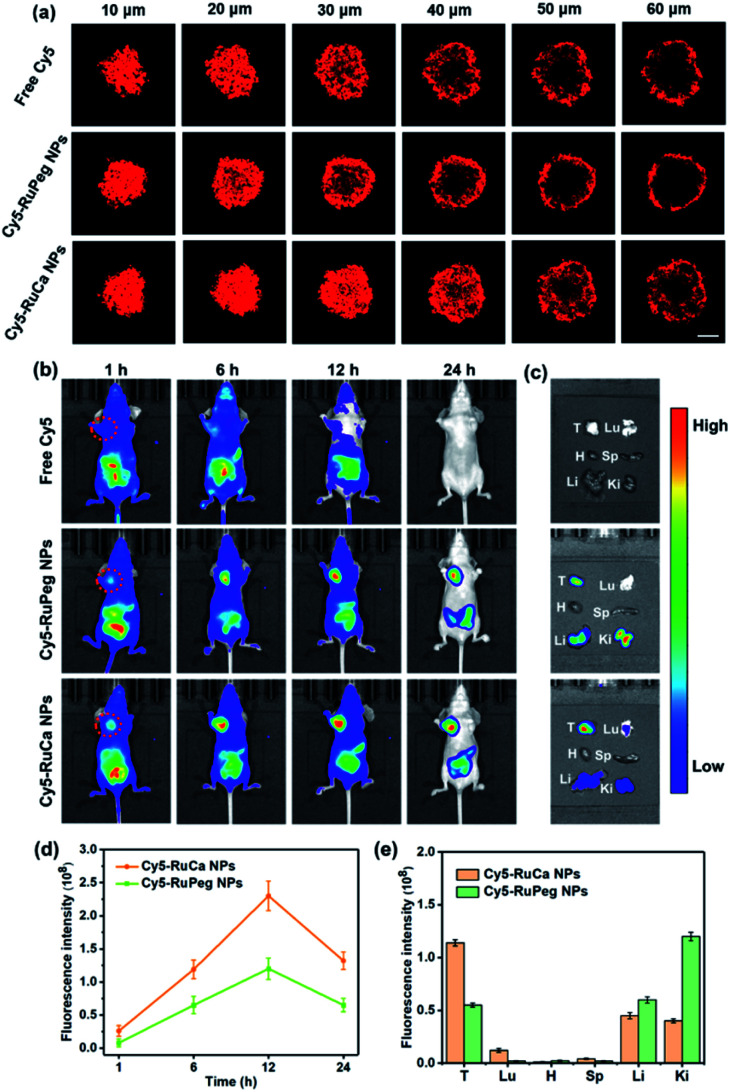
(a) *In vitro* penetration of Cy5 into MCTSs after incubation with free Cy5, Cy5-RuCa or Cy5-RuPeg NPs under hypoxic conditions. Scale bar: 100 μm. (b) Fluorescence images of nude mice bearing MDA-MB-231 tumor after intravenous injection of Cy5, Cy5-RuPeg NPs and Cy5-RuCa NPs. Images were taken at 1, 6, 12, and 24 h after injection. The dashed circle indicates the tumor. (c) Fluorescence images of major organs and the tumor. Images were taken at 24 h post injection. (d) Semi-quantitative analysis of Cy5-RuPeg NPs and Cy5-RuCa NPs in MDA-MB-231 tumors after intravenous injection. (e) Semi-quantitative analysis of Cy5-RuPeg NPs and Cy5-RuCa NPs in major organs and tumor tissues from MDA-MB-231 tumor-bearing mice at 24 h post-injection. Mean ± SD, *n* = 3.

In order to investigate the penetration behavior of the prepared nanoparticles in a model closer to the real tumors, MDA-MB-231 3D multicellular tumor spheroids (MCTSs) have been used for the study. After 12 h incubation of MCTSs with free Cy5, Cy5-RuCa or Cy5-RuPeg NPs (equal concentration of Cy5) under hypoxic conditions, MCTSs were exposed to 660 nm laser irradiation. After another 6 h incubation, CLSM was utilized to observe the sample penetration within MCTSs. As shown in Fig. 6a, the bright red fluorescence of Cy5-RuPeg NPs rapidly decreased with increasing depth, illustrating the penetration depth of Cy5-RuPeg NPs. Remarkably, Cy5-RuCa NPs exhibited the best penetration capability as visualized by the wide distribution of red fluorescence in most regions of MCTSs, which can be ascribed to the CAIX-mediated active transport and the photodegradation of RuCa under irradiation.

### NIR fluorescence imaging *in vivo*

The *in vitro* results encouraged us to further study the biodistribution and accumulation of Cy5-RuCa and Cy5-RuPeg NPs in nude mice bearing MDA-MB-231 tumor. After the mice were injected with Cy5-RuCa or Cy5-RuPeg NPs through the tail vein, fluorescence imaging was used to visualize the biodistribution of nanoparticles in mice through the detection of Cy5 fluorescence at different time points. As shown in Fig. 6b and d, the fluorescence signals of Cy5-RuCa NPs were gradually increased in tumors with time, and an intense peak was also observed at 12 h, indicating the significant retention ability of Cy5-RuCa NPs in the tumor due to its dual-targeting feature through CAIX targeting and the EPR effect. Besides, the fluorescence intensity of Cy5-RuCa NPs was found to be maintained at the tumor site for 24 h after injection, suggesting the long retention time of Cy5-RuCa NPs in the tumor. This could be attributed to the good stability and slow clearance of the vehicle. As expected, Cy5-RuPeg NPs showed relatively weak fluorescence intensity in tumors as compared to Cy5-RuCa NPs. The mice were sacrificed after 24 h, and their main organs and tumors were collected for fluorescence imaging. Compared with the main organs, the tumor treated with Cy5-RuCa NPs exhibited much stronger fluorescence intensity after dissection (Fig. 6c and e), further proving the targeting specificity of Cy5-RuCa NPs toward the tumor.

### *In vivo* synergistic therapy

**Fig. 7:**
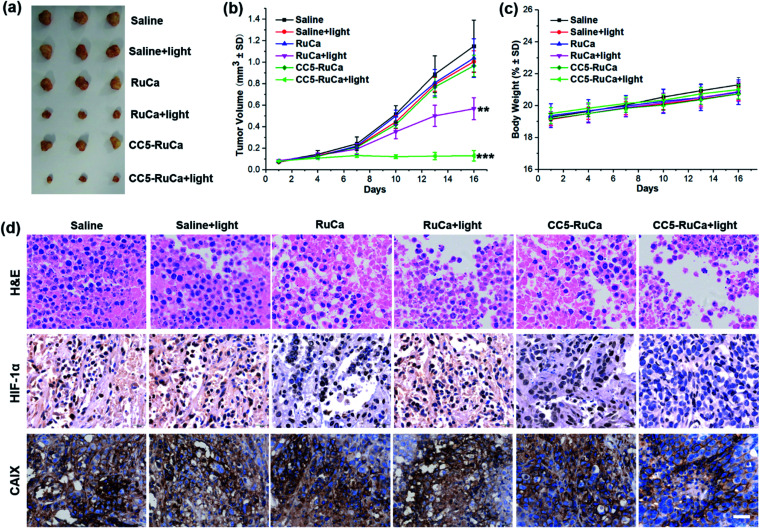
(a) Gross solid tumor images from sacrificed mice after treatments. (b) The tumor growth curves with different treatments (**p < 0.01, ***p < 0.001) *versus* PBS (two-tailed Student's *t*-test). Error bars indicate standard deviation (SD; *n* = 3). (c) Body weight changes in the course of treatments. (d) H&E stained tumor sections and representative photomicrographs of tumor slices immunostained with HIF-1α and CAIX antibody. Scale bar: 40 μm.

The *in vivo* antitumor efficacy of RuCa and CC5-RuCa was evaluated in nude mice bearing MDA-MB-231 tumors. The mice were randomly divided into six groups, then RuCa and CC5-RuCa were administered through the tail vein at a dose of 15.0 mg kg^−1^ on the first, sixth and eleventh days, followed by 660 nm (0.2 W cm^−2^, 10 min) laser irradiation on the tumors at 12 h post injection (Fig. S25, ESI†). As shown in Fig. 7a and b and S26 (ESI†), the tumor growth of the MDA-MB-231 cell xenograft suppressed by the saline plus light group is only 2.56%, indicating that the laser has a negligible influence on tumor growth. Meanwhile, RuCa and CC5-RuCa in the absence of laser irradiation showed similar tumor growth profiles to that of the saline plus light group, further confirming the low dark toxicity of RuCa and CC5-RuCa. In contrast, once irradiation was performed, RuCa and CC5-RuCa exhibited remarkable tumor inhibition effects with 54.2% and 87.5% tumor-suppression-rates, respectively. Impressively, the tumor growth was totally inhibited after the second administration of CC5-RuCa. Moreover, no significant difference among body weights of the mice was observed in any of the groups (Fig. 7c), indicating good biocompatibility and low systemic toxicity of the micelles. The blood biochemical analysis indicated that treatment of CC5-RuCa NPs had no effect on parameters of liver and kidney functions in mice, further confirming the biosafety of CC5-RuCa NPs (Fig. S27, ESI†).

After the treatment, the mice were sacrificed, and all primary tumors were collected, pictured, fixed, and sectioned for the standard hematoxylin and eosin (H&E) staining. As shown in Fig. 7d, apparent cellular necrosis or apoptosis in the tumor section was observed in the CC5-RuCa plus light treated group, while the cells in the other groups were seen to largely exhibit the normal morphology and proliferation patterns. Besides, HIF-1α and CAIX immunostaining were performed to evaluate the changes of tumor hypoxia status after different treatments.^54^ As shown in Fig. 7d, the expressions of HIF-1α and CAIX proteins were down-regulated in the CC5-RuCa and CC5-RuCa plus light treated groups, possibly attributed to the function of YC-1. In contrast, the expressions of HIF-1α and CAIX proteins in the RuCa plus light treated group were up-regulated due to the consumption of the oxygen by PDT.

## Discussion

Based on the reduction of azo groups by azoreductase within the hypoxic tumor microenvironment, we have developed a hypoxia-activated self-immolative prodrug containing a HIF-1α inhibitor and a HDAC inhibitor for the first time. HPLC profiles showed that CC5 can take advantage of the tumor-associated microenvironment to release CI-994 and YC-1 under hypoxic and acidic conditions. In contrast, negligible degradation of CC5 was observed under normoxia, implying that CC5 could be selectively degraded to release the cytotoxic agents under hypoxia. The *in vitro* cytotoxic activities of CC5 toward five cell lines have been investigated. As expected, CC5 displayed greatly improved cytotoxic activity under hypoxia compared to that under normoxia with HF (hypoxia factor: the toxicity under normoxia *vs.* hypoxia) values ranging from 1.4 to 30.8. Besides, the *in vitro* apoptosis study revealed that CC5 could induce a 2.3-fold increased incidence of early to late stage apoptosis toward MDA-MB-231 cells compared with that under normoxia, further confirming the potent cytotoxicity of CC5 against cancer cells under hypoxic conditions.

Encouraged by the ideal *in vitro* results, we further evaluated the *in vivo* therapeutic efficacy of CC5 on the MDA-MB-231 cell xenograft mice. The results showed that CC5 efficiently inhibited the tumor growth in a dose-dependent manner, with 59.3% and 70.8% reduction in tumor weight at concentrations of 2.4 and 4.5 mg kg^−1^, respectively. Despite the fact that CC5 exhibited potent *in vivo* anticancer activity, there is no advantage for CC5 in comparison with DOX. Moreover, the body weights of the mice decreased after the adminstration of CC5, demonstrating the low biocompatibility and potent systemic toxicity of CC5. According to our assumption, the unsatisfactory *in vivo* performance of CC5 may be attributed to the following reasons. First, a small molecule agent like CC5 usually has a short blood circulation time and low tumor accumulation due to its intrinsic limitations such as poor physicochemical properties and nonspecific tumor targeting ability. Second, although a part of CC5 is able to reach the tumor tissue, oxygen concentration in the exterior of the tumor may not be low enough to induce the degradation of CC5.

In order to improve the bioavailability and maximize the *in vivo* therapeutic efficacy of CC5, it was further encapsulated with a photolabile amphiphilic ruthenium complex-derived polymer. The resulting nanosystem can not only be used as a controlled-release delivery system for CC5 targeting delivery, but also reduce the oxygen concentration at tumor sites *via* generating ROS upon irradiation. The prepared CC5-RuCa NPs were characterized by TEM, DLS, UV-vis spectra and HPLC. Upon 660 nm laser irradiation, the MLCT band of RuCa gradually red-shifted from 553 nm to *ca.* 650 nm, indicating the ligand dissociation of RuCa. The result was further confirmed by the TEM images as revealed by the rupture of the spherical nanoparticles. This study provides a foundation for the light-controlled CC5 release at the tumor tissue. *In vitro* cytotoxicity results showed that CC5-RuCa NPs exhibited considerable photocytotoxicity, especially under hypoxic conditions, reflecting the potential synergistic effect of PDT and hypoxia-activated chemotherapy. The biodistribution and accumulation profiles revealed the long circulation time and tumor targeting capability of the nanoparticles *in vivo*. Finally, the *in vivo* antitumor efficacy of CC5-RuCa NPs was evaluated. Remarkably, CC5-RuCa NPs with good biocompatibility and low systemic toxicity exhibited a much higher tumor-suppression-rate in comparison with RuCa and CC5, which is possibly attributed to the synergistic effect of PDT and hypoxia-activated chemotherapy.

In previous literature reports, the strategy of introducing PDT therapy into hypoxia-activated systems has been demonstrated to be an effective and promising method for tumor therapy. However, the hypoxia-activated prodrugs currently studied were very limited to a few agents like AQ4N and TPZ. Therefore, it is meaningful to develop the novel hypoxia-activated prodrug CC5 with synergistic anticancer effects by combining a hypoxia-activated target drug with a hypoxia-targeted inhibitor. Moreover, the photolabile ruthenium complex-derived polymer was used as a photosensitizer to generate ROS to kill the tumor cells and boost the release of the prodrug CC5 with enhanced intratumoral penetration in the severe hypoxia region inside the tumor tissue. However, the drug loading capacity of CC5-RuCa NPs is only about 14.5%, which is relatively low compared with other drug delivery systems. In addition, the physical encapsulation of CC5 *via* noncovalent interactions may suffer from instability and uncontrolled release. Moreover, the complicated preparation process of CC5-RuCa NPs may lead to poor reproducibility on a large scale. Hence, further research will be focused on covalent conjugation of the hypoxia-activated prodrug to the photolabile amphiphilic ruthenium complex-derived polymer, which is expected to have the potential to overcome the current limitations.

## Conclusion

In summary, we have developed a bifunctional hypoxia-activated prodrug (CC5) which could make full use of the advantages of the tumor microenvironment to selectively release the cytotoxic agent (CI-944) and HIF-1α inhibitor (YC-1) under hypoxic and acidic conditions. To maximize the therapeutic efficacy of CC5, a photo-responsive ruthenium complex-derived micelle (CC5-RuCa) loaded with CC5 was prepared for light and tumor microenvironment-controlled multistage drug-release. Under NIR irradiation, CC5-RuCa can generate ROS to kill the cancer cells and release CC5 in the exterior of the tumor. Subsequently, the released prodrug CC5 can penetrate deep into the severe hypoxia region inside the tumor tissue to facilitate the tumor therapy. *In vivo* studies reveal that CC5-RuCa with preferential tumor accumulation exhibit highly efficient tumor regression (87.5%) through the synergistic effect of PDT and hypoxia-activated chemotherapy. Consequently, our research has provided a precise and promising platform for constructing light-controlled nanocarriers that can selectively identify hypoxic cancer cells and combine hypoxia-activated prodrugs with PDT to promote the effect of cancer treatments.

## Data availability

The data that support the findings of this study are available from the corresponding author upon request.

## Author contributions

B. Z., Z. X., J. Z. and S. G. developed the concept and design of the project. Synthesis and physico-chemical characterization steps were performed by B. Z., Z. X. and Z. L. Drug-release profiles were obtained by B. Z. Cell assays were performed by W. Z. All authors contributed to the analysis and interpretation of the data. The manuscript was written by B. Z. and Z. X. and revised by J. Z. and S. G. All authors have given approval for the final version of the manuscript.

## Conflicts of interest

There are no conflicts to declare.

## Supplementary Material

SC-012-D1SC01888D-s001
