# Supplementary material for: A light-controlled multi-step drug release nanosystem targeting tumor hypoxia for synergistic cancer therapy
Source: Chem Sci. 2021 Aug 3;12(35):11810–20. doi: 10.1039/d1sc01888d (PMC8442699; doi:10.1039/d1sc01888d)
Supplement: SC-012-D1SC01888D-s001 [file SC-012-D1SC01888D-s001.pdf]

Electronic Supplementary Material (ESI) for Chemical Science.
This journal is © The Royal Society of Chemistry 2021

A light-controlled multi-step drug release nanosystem targeting tumor hypoxia for synergistic cancer therapy

Bin Zhang,‡[ab] Zichen Xu,‡[ab] Wen Zhou,[ab] Zhikun Liu,[ab] Jian Zhao*[ab] and Shaohua Gou*[abc]

[a] *Jiangsu Province Hi-Tech Key Laboratory for Biomedical Research, Southeast University, Nanjing 211189, China*

[b] *Pharmaceutical Research Center and School of Chemistry and Chemical Engineering, Southeast University, Nanjing 211189, China*

[c] *Nanjing Junruo Institute of Biomedicine, Nanjing 211100, China*

E-mail: zhaojianzhaokuan@163.com (Jian Zhao); 2219265800@qq.com (Shaohua Gou)

Table of Contents

Experimental section…………………………………………………….............. S4-S14

1. Materials and instruments
2. Synthesis of CC5 and RuCa
3. Hypoxia-induced breakdown of CC5
4. Photocleavage of RuCa
5. The $^1O_2$ generation
6. Preparation and characterization of RuCa and CC5-RuCa nanoparticles
7. Preparation of Cy5-RuCa and Cy5-RuPeg nanoparticles
8. *In vitro* cytotoxicity evaluation
9. Apoptosis analysis
10. Western blot analysis
11. *In vivo* antitumor activity of CC5
12. Cellular internalization evaluation
13. Intracellular ROS production
14. 3D multicellular tumor spheroids
15. *In vivo* biodistribution
16. *In vivo* antitumor efficacy of CC5-RuCa
17. Histological analysis

Supplementary tables and figures…………….……….…….….……..... S15-S28

Table S1. IC_{50} values of CI-994, CC4 and CC5 against different cells under normoxia.

Table S2. IC_{50} values of CI-994, CC4 and CC5 against different cells under hypoxia.

Table S3. Cytotoxicity of RuCa and CC5-RuCa against MDA-MB-231 cells with and without light irradiation under normoxic or hypoxic condition.

Figure S1-S10. ^1H NMR, ^{13}C NMR and HR-MS spectra of CC5 and its intermediates.

Figure S11. HPLC diagrams of CC5 under normoxic or hypoxic microenvironment at pH 5.0.

Figure S12. Tumor weight of the mice from each group after treated with CC5 and DOX.

Figure S13. Body weight of the excised mice of each group after treated with CC5 and DOX.

Figure S14-S19. ^1H NMR or HR-MS spectra of RuCa and its intermediates.

Figure S20. ^1H NMR spectrum of RuPeg.

Figure S21. HPLC diagrams of CC5-RuCa.

Figure S22. The standard curve of CC5 determined by HPLC with a UV detection at 254 nm.

Figure S23. TEM images of RuCa NPs under different 660 nm laser irradiation time.
Figure S24. Absorption spectra of ABDA for RuCa with or without 660 nm light irradiation.
Figure S25. Images of the mice after different treatments for 16 days.
Figure S26. Tumor weight of the mice from each group at the end of the observation period.
Figure S27. Serum chemistry analysis of mice serum that receiving *i.v.* injection of RuCa or CC5-RuCa NPs.
General synthetic procedures of CC5 and RuCa……….………..……..... S29-S30
Scheme S1. Preparation of CC5.
Scheme S2. Preparation of RuCa.
References…………………..……………..……………..…………..……..... S31

Experimental section

1. Materials and instruments

All chemicals and solvents were of analytical reagent grade and used without further purification. $RuCl_3 \cdot xH_2O$ (99.9%) was purchased from Alfa Aesar. Acetazolamide and HO-PEG$_{4000}$-N$_3$ were purchased from Aladdin Biochemical Technology Co., Ltd. (Shanghai, China) and Shanghai Ponsure biotechnology Co. Ltd. (Shanghai, China), respectively. YC-1 and CI-994 were prepared according to our and others reports.[1-3] The GAPDH, p21 and HIF-1α antibodies were purchased from Santa Cruz Biotechnology. Ac-histone H3 antibodies were purchased from Calbiochem. All cancer cell lines were obtained from Jiangsu KeyGEN BioTECH company (China). ^{1}H NMR and ^{13}C NMR spectra were recorded in DMSO-d_6 on a Bruker 300 or 600 MHz spectrometer. Mass spectra were measured by an Agilent 6224 ESI/TOF MS instrument. UV-vis absorption spectra were recorded on a Shimadzu UV2600 instrument. High performance liquid chromatography (HPLC) was performed on a Waters e2695 system equipped with a Symmetry C18 column (250 × 4.6 mm, 5 mm).

2. Synthesis

2.1. Synthesis of CC1

4-aminobenzoic acid (8.00 g, 58.4 mmol) was dissolved in 60 mL of MeOH, 15 mL concentrated sulfuric acid was added and refluxed at 80 °C for 5 h, aqueous NaOH solution was added dropwise to adjust pH = 3. The mixture was concentrated under vacuum and washed with water to obtain colorless solid (7.91 g, yield 89.5%). ^{1}H NMR (300 MHz, Methanol-d_4) δ 7.74 (d, J = 8.7 Hz, 2H), 6.64 (d, J = 8.7 Hz, 2H), 3.82 (s, 3H) ppm. ESI-MS (m/z): calcd for $C_8H_9NO_2$ $[M+H]^+$: 152.0667, found: 152.0689.

2.2. Synthesis of CC2

CC1 (4.00 g, 26.0 mmol) was suspended in 20 mL of CH_2Cl_2. Oxone (23.9 g, 39.0 mmol), dissolved in 200 mL of water, was added to the mixture and stirred fiercely overnight at room temperature. The organic phase was separated, and the

aqueous phase was extracted with 100 mL of CH_2Cl_2. The combined organic phases were washed with 1 N HCl (100 mL), saturated aq $NaHCO_3$ (100 mL), and water (100 mL), respectively, then dried over $MgSO_4$ and concentrated, residual solid was recrystallized from CH_2Cl_2 and the product was obtained as green solid (2.33 g, yield 54.3%). ^{1}H NMR (300 MHz, DMSO-d_6) δ 8.30 (d, J = 8.4 Hz, 2H), 8.07 (d, J = 8.5 Hz, 2H), 3.93 (s, 3H) ppm.

2.3. Synthesis of CC3

A solution of CI-994 (0.30 g, 1.11 mmol) in 5 mL DMF was degassed with a nitrogen stream for 15 min. Then, CC2 (0.73 g, 4.44 mmol) was added. The mixture was stirred at 80 °C for 24 h, and the solvent was removed under reduced pressure. The residue was purified on silica gel column eluted DCM/MeOH (10:1) to give the desired product as a red brown solid (1.07 g, yield 23.2%). ^{1}H NMR (300 MHz, Methanol-d_4) δ 10.75 (s, 1H), 10.24 (s, 1H), 8.33 (d, J = 8.3 Hz, 1H), 8.17 (d, J = 8.4 Hz, 2H), 8.00 (dd, J = 12.9, 8.6 Hz, 4H), 7.80 (dd, J = 18.0, 8.4 Hz, 3H), 7.65 (t, J = 7.6 Hz, 1H), 7.34 (t, J = 7.6 Hz, 1H), 3.91 (s, 3H), 2.10 (s, 3H) ppm. ESI-MS (m/z): calcd for $C_{23}H_{20}N_4O_4$ $[M-H]^-$: 415.1485, found: 415.1535.

2.4. Synthesis of CC4

CC3 (0.20 g, 0.48 mmol) was dissolved in 3 mL of DMF, LiOH (0.06 g, 1.44 mmol) was dissolved in water and added to solution. Mixture was stirred at room temperature for 3 h. The solvent was evaporated under reduced pressure and 1 N HCl was added dropwise to adjust pH = 3. The solid was collected by suction filtration, dried, and the residue was purified on silica gel column eluted DCM/MeOH (2:1) to give the desired product as a red solid (0.08 g, 78.2%). ^{1}H NMR (600 MHz, DMSO-d_6) δ 10.78 (s, 1H), 10.31 (s, 1H), 8.32 (d, J = 8.0 Hz, 1H), 8.14 (d, J = 7.9 Hz, 2H), 7.99 (t, J = 7.4 Hz, 4H), 7.83 (d, J = 8.1 Hz, 1H), 7.78 (d, J = 8.2 Hz, 2H), 7.64 (t, J = 7.7 Hz, 1H), 7.35 (t, J = 7.6 Hz, 1H), 2.11 (s, 3H) ppm. ESI-MS (m/z): calcd for $C_{22}H_{18}N_4O_4$ $[M-H]^-$: 401.1328, found: 401.1356.

2.5. Synthesis of CC5

YC-1 (0.12 g, 0.40 mmol), TBTU (0.19 g, 0.60 mmol) and TEA (0.06 g, 0.60

mmol) were dissolved in 5 mL dry DMF, and stirred at room temperature for 5 min, then CC4 (0.16 g, 0.40 mmol) was added. The mixture was stirred at 45°C overnight, the solvent was evaporated under reduced pressure, the residue was purified on silica gel column eluted DCM/MeOH (8:1) to give the desired product as a red solid (0.09 g, 31.8%). ^{1}H NMR (600 MHz, DMSO-d_6) δ 10.78 (s, 1H), 10.29 (s, 1H), 8.33 (d, J = 8.2 Hz, 1H), 8.13 (dd, J = 12.1, 8.3 Hz, 2H), 7.99 (dd, J = 8.5, 5.5 Hz, 3H), 7.83 (d, J = 9.3 Hz, 1H), 7.76 (dd, J = 19.0, 8.6 Hz, 3H), 7.64 (t, J = 8.4 Hz, 1H), 7.45 (t, J = 7.6 Hz, 1H), 7.42–7.29 (m, 4H), 7.29–7.21 (m, 5H), 6.97 (d, J = 3.3 Hz, 1H), 6.48 (d, J = 3.2 Hz, 1H), 5.71 (s, 2H), 4.51 (s, 2H), 2.11 (s, 3H) ppm. ^{13}C NMR (150 MHz, DMSO-d_6) δ 171.17, 164.02, 161.35, 149.32, 149.08, 146.91, 140.80, 137.74, 137.30, 135.44, 133.23, 130.11, 129.06, 128.05, 127.70, 127.40, 126.40, 125.67, 124.01, 123.39, 122.67, 122.16, 121.80, 121.45, 120.72, 119.82, 113.45, 112.54, 110.74, 110.03, 108.45, 103.03, 60.84, 58.51, 22.56 ppm. ESI-MS (m/z): calcd for $C_{41}H_{32}N_6O_5$ $[M-H]^-$: 687.2434, found: 687.3489.

2.6. Synthesis of CA-1

CA-1 was prepared according to the literature method.[4]

2.7. Synthesis of HA-1

6-Heptynoic acid (0.63 g, 5.0 mmol) in dry CH_2Cl_2 (15 mL) was stirred at 0 °C and oxalyl chloride (0.95 g, 7.5 mmol) was dropped into the mixture and stirred at room temperature for 6 h. After the reaction, the solvent and excess oxalyl chloride were evaporated under reduced pressure to offer compound HA-1.

2.8. Synthesis of CA-2

Compound HA-1 (5.0 mmol) was slowly added to the mixture of CA-1 (0.72 g, 4.0 mmol) and triethylamine (0.61 g, 6.0 mmol) at 0 °C. The mixture was kept stirring at room temperature overnight. After the reaction, the solvent was evaporated under reduced pressure, and the crude product was purified by chromatography on silica gel eluted with DCM/MeOH (30 : 1 v/v) to offer compound CA-2 as a white solid (0.96 g, yield 83.3%). ^{1}H NMR (600 MHz, DMSO-d_6) δ 13.00 (s, 1H), 8.32 (s, 2H), 2.78 (t, J = 2.6 Hz, 1H), 2.55 (t, J = 7.4 Hz, 2H), 2.20–2.17 (m, 2H), 1.73–1.68 (m, 2H),

1.50–1.45 (m, 2H) ppm. HR-MS (m/z): calcd for $C_9H_{13}N_4O_3S_2$ $[M+H]^+$: 289.0424, found: 289.0423.

2.9. Synthesis of CA-PEG

To a solution of CA-2 (28.8 mg, 0.1 mmol) in MeOH (5 mL), HO-PEG_{4000}-N_3 (400 mg, 0.1 mmol) and catalytic amount of $CuSO_4 \cdot 5H_2O$ were added. After the resulting mixture was stirred for 5 min under nitrogen atmosphere, catalytic amount of sodium ascorbate was added, then the mixture was kept stirring at room temperature overnight. Removal of the organic solvent by evaporation under reduced pressure yielded crude products, the crude product was purified by chromatography on silica gel eluted with DCM/MeOH (20 : 1 v/v) to offer compound CA-PEG as a white solid (0.30 g, yield 81.5%).

2.10. Synthesis of $Ru(Biq)_2Cl_2$

$Ru(Biq)_2Cl_2$ was synthesized via a literature way.[5]

2.11. Synthesis of $[Ru(Biq)_2(Cya)_2](PF_6)_2$

$[Ru(Biq)_2(Cya)_2](PF_6)_2$ was obtained by a method similar to the synthesis of $[Ru(Biq)_2(CH_3CN)_2](PF_6)_2$.[5] $Ru(Biq)_2Cl_2$ (1.0 g, 1.4 mmol) and $AgPF_6$ (0.46 g, 2.9 mmol) were dissolved in 1:1 ethanol/H_2O mixture (80 mL). The solution was degassed and heated under reflux overnight in a nitrogen atmosphere. The solution was cooled and filtered to remove AgCl. The solvent of the reaction was reduced to ~40 mL by evaporation under reduced pressure. Then, an aqueous solution of KPF_6 was added. The precipitate was filtered, washed with H_2O, and dried to obtain a blue solid. The blue solid (0.94 g, 1.0 mmol) and 4-cyanophenol (0.26 g, 2.2 mmol) were mixed in acetone (80 mL). The mixture was stirred under reflux overnight in the dark in a nitrogen atmosphere. After that, the solvent was evaporated under reduced pressure. Then, the obtained crude product was purified through column chromatography on silica gel eluted with DCM/MeOH (20 : 1 v/v) to yield the product as a red solid (809 mg, 67.9%). ^1H NMR (600 MHz, DMSO-d_6) δ 9.59 (s, 2H), 8.93 (d, J = 8.6 Hz, 2H), 8.60 (d, J = 7.6 Hz, 2H), 8.47 (d, J = 8.0 Hz, 2H), 8.47–8.19 (m, 8H), 8.10 (t, J = 7.0 Hz, 2H), 8.06 (d, J = 8.0 Hz, 2H), 7.58 (t, J = 7.5 Hz,

2H), 7.48 (d, *J* = 8.3 Hz, 4H), 6.95 (d, *J* = 8.7 Hz, 6H), 6.68 (d, *J* = 8.4 Hz, 2H) ppm. HR-MS (m/z): calcd for $C_{50}H_{35}N_6O_2Ru$ $[M+H]^+$: 853.1680, found: 853.1796.

2.12. Synthesis of RuCa

To a solution of 4-nitrophenyl chloroformate (12.0 mg, 0.059 mmol) and TEA (15.5 mg, 0.12 mmol) in THF (3 mL), a solution of CA-PEG (240 mg, 0.059 mmol) in THF (3 mL) was added dropwise in 20 min at 0 ℃. The reaction mixture was stirred in the dark at room temperature for 5 h. After removal of the solvents, the obtained crude product was redissolved in anhydrous DMF (3 mL), and added to a solution of $[Ru(Biq)_2(Cya)_2](PF_6)_2$ (25.6 mg, 0.03 mmol) and TEA (15.5 mg, 0.12 mmol) in DMF at 0 °C. The reaction mixture was stirred for overnight at room temperature. The final clear organic solution was dialyzed against water to remove unreacted CA-PEG, complex Ru and TEA within a dialysis bag (molecular weight cutoff: 5000 Da) for 24 h. After lyophilization, the target intermediate polymer, RuCa, was collected (yield: 75%).

3. Hypoxia-induced breakdown of CC5

A PBS (pH = 7.4 or 5.0, 1% DMSO) solution of CC5 containing rat liver microsomes (20 μL, 20 mg/mL) (Shanghai Bioroot Biological Technology Co., Ltd, China) and nicotinamide adenine dinucleotide phosphate disodium salt (NADPH) (100 μM) were incubated under normoxic (20% O_2, 5% CO_2, and N_2 75%) or hypoxic (1% O_2, 5% CO_2, and N_2 94%) condition at 37 °C. The hypoxia-induced breakdown of CC5 at different times was evaluated by HPLC using a mixture of solvent acetonitrile/water at the flow rate of 1.0 mL/min with peak detection at 254 nm under UV.

4. Photocleavage of RuCa

The absorption spectra of RuCa were recorded on a Shimadzu UV2600 UV-vis spectrophotometer. The electronic spectra of RuCa dissolved in DMF/H_2O (1/99, v/v) were obtained at different times with laser irradiation (660 nm, 30 mW/cm^2). Besides, the solutions of RuCa after irradiated 0, 10, and 30 min were also determined by HPLC with a UV detection at 254 nm.

5. The $^1O_2$ generation

RuCa in H_2O (50 μg/mL) was mixed with the same volume of ABDA aqueous solution (100 μM). The absorption spectra were recorded every 10 s under 660 nm LED irradiation (30 mW/cm^2). For the negative control without irradiation, the nanoparticles (25 μg/mL) in water containing ABDA (50 μM) were recorded every 10 min for 50 min.

6. Preparation and characterization of RuCa and CC5-RuCa nanoparticles

For the preparation of CC5-loading nanoparticles, RuCa (20 mg) and CC5 (4 mg) were dissolved in DMSO (2 mL) and stirred for 1 h in dark. Then, the solution was added dropwise into 10 mL deionized water with rapid stirring. After stirring for 3 h, the solution was dialyzed against deionized water for one day to remove the organic solvents and free CC5 using a dialysis tube (MWCO: 5000 Da). In the dialysis process, deionized water was replaced approximately every 6 h. The solution was then lyophilized for use. The method of preparing RuCa nanoparticles was the same as that of CC5-RuCa. The morphology of RuCa and CC5-RuCa was determined by the transmission electron microscope (TEM) (Tecnai G20). UV/vis spectra with different wavelengths were recorded on a Shimadzu spectrophotometer (UV2600). Drug-loading content and drug-loading efficacy were determined by redissolving CC5-RuCa in DMSO, and then testing the content of CC5 by HPLC with a UV detection at 254 nm. The drug-loading content and drug-loading efficacy of CC5 were calculated as follows: Loading content = (WT−WF)/WNP × 100%, Encapsulation efficiency = (WT−WF)/WT × 100%, where WT is the total weight of CC5 fed, WF is the weight of non-encapsulated free CC5, and WNP is the weight of nanoparticles.

7. Preparation of Cy5-RuCa and Cy5-RuPeg nanoparticles

RuCa (5 mg) and Cy5 (1.0 mg) were dissolved in 1 mL of anhydrous DMSO and stirred at room temperature for 1 h. Then 3 mL of distilled water was added dropwise within 10 min and stirred for 1 h in dark. After that, the mixture was dialyzed against distilled water with a dialysis bag (MWCO: 5000 Da) for 24 h. The solution was then

lyophilized and dissolved in DMSO for content determination. The Cy5 content in Cy5-RuCa was determined by absorbance at 640 nm.

8. *In vitro* cytotoxicity evaluation

Human lung (A549), breast (MCF-7 and MDA-MB-231), colon (HCT-116), gastric (SGC7901) and cervical (HeLa) cancer cell lines along with human umbilical vein endothelial cell line (HUVEC) were maintained in the logarithmic phase at 37°C in a 5% carbon dioxide atmosphere using the following monolayer culture media containing 10% fetal bovine serum (FBS), 100 mg/mL of penicillin and 100 mg/mL of streptomycin. The growth inhibitory effect towards human cell lines was evaluated by means of MTT assay. Briefly, 1×10^5 cells/well, dependent upon the growth characteristics of the cell line, were seeded in 96-well plates in DMEM medium with 10% FBS and then incubated at 37 °C in a humidified atmosphere of 5% CO_2/95% air. After 24 h, the medium was removed and replaced with a fresh one containing the compound to be studied at the appropriate concentration. Triplicate cultures were established for each treatment. After incubation with the target compounds under normoxic condition (20% O_2, 5% CO_2 and 75% N_2, at 37 °C) or hypoxic condition (1% O_2, 5% CO_2 and 94% N_2, at 37 °C) for 72 h, the cells was treated with 10 μL of a 5 mg·mL^{-1} MTT for 5 h additional incubation. The medium was thrown away and replaced by 100 μL DMSO. The inhibition of cell growth induced by the tested complexes was detected by measuring the absorbance of each well at 570/630 nm using enzyme labeling instrument.

9. Apoptosis analysis

MDA-MB-231 cells were seeded at the density of 2×10^6 cells/mL of the DMEM medium with 10% FBS on 6-well plates to the final volume of 2 mL. The plates were incubated for overnight and then treated with different concentrations of CI-994 and CC5 for 48 h. Briefly, after incubation under normoxic condition (20% O_2, 5% CO_2 and 75% N_2, at 37 °C) or hypoxic condition (1% O_2, 5% CO_2 and 94% N_2, at 37 °C) for 48 h, cells were collected and washed with PBS twice, and then resuspended cells in 1 × Binding Buffer (0.1 M Hepes/NaOH (pH 7.4), 1.4 M NaCl, 25 mM $CaCl_2$) at a

concentration of 1×10^6 cells/mL. The cells were stained with 5 μL of FITC Annexin V (BD, Pharmingen) and 5 μL propidium iodide (PI) staining using annexin-V FITC apoptosis kit followed; 100 μL of the solution was transferred to a 5 mL culture tube and incubated for 30 min at room temperature (25°C) in the dark. The apoptosis ratio was quantified by system software (Cell Quest; BD Biosciences).

10. Western blot analysis

MDA-MB-231 cells were lysed in cell lysis buffer containing PMSF for 30 min at 4 °C. Lysates were collected by centrifugation at 13000 rpm for 20 min at 4 °C. Proteins from cell lysates were separated on the SDS-PAGE and transferred onto polyvinylidine difluoride (PVDF) membrane (Amersham Biosciences). The membrane was blocked with PBST containing 5% non-fat dry milk for 1 h and further incubated with monoclonal anti-human HIF-1α antibody (Santa Cruz Biotechnology, USA), anti-p21 antibody (Santa Cruz Biotechnology, USA) or anti-Ac-histone H3 antibody (Calbiochem, Germany) overnight at 4 °C under gentle shaking. After that, the membrane was incubated with the secondary antibody (1:2000) for 1 h at RT (25 °C). Protein blots were detected with chemiluminescence reagent (Thermo Fischer Scientifics Ltd.). Anti-GAPDH antibody was used as loading control.

11. *In vivo* antitumor activity of CC5

The *in vivo* cytotoxic activity of CC5 was investigated on the MDA-MB-231 cell xenograft mice model. Five-week-old female BALB/c nude mice (18-22 g) were purchased from Shanghai Ling Chang biotechnology company (China). Tumors were induced by a subcutaneous injection in their right armpit region of 10^7 cells in 0.1 mL of sterile PBS. When the tumors reached a volume of 100-150 mm^3 in all mice, animals were randomly divided into four groups, and started on the second day. The first group was injected with an equivalent volume of 5% dextrose via a tail vein as the vehicle control mice. No. 2 group was treated with DOX at doses of 2 mg/kg. No. 3 and No. 4 groups were treated with CC5 at the doses of 2.4 or 4.5 mg/kg. DOX was dissolved in vehicle. CC5 was dissolved in a small amount of DMF, and then diluted with Tween 80 and 5% dextrose injection. The final solution contains DMF: Tween

80: 5% dextrose injection = 10: 2: 88. Tumor volume and body weight were recorded every other day after drug treatment. All mice were sacrificed after 4 weeks of treatment and the tumor volumes were measured with electronic digital calipers and determined by measuring length (A) and width (B) to calculate volume ($V = AB^2/2$). The animal procedure followed the guidelines of the Institutional Animal Care and Use Committee at KeyGEN BioTECH Co. Ltd (laboratory accreditation number: SYXK-2017-0015). BALB/c nude mice were purchased from Shanghai SLAC Laboratory Animal Co. Ltd. All surgical interventions and postoperative animal care procedures were approved by the Experimental Animal Ethics Committee of Southeast University (Nanjing, China).

12. Cellular internalization evaluation

MDA-MB-231 cells were cultured overnight in the confocal laser dish or six-well tissue culture plate. The medium was replaced with fresh one containing Cy5-RuPeg or Cy5-RuCa at 0.5 μg mL^{-1} of Cy5. Then the cells were incubated for 4 h under hypoxia. The confocal fluorescence images were obtained via CLSM. For staining of cell nuclei, 4',6-diamidino-2-phenylindole (blue color) was employed for 15 min of incubation and the cells were then observed via CLSM.

13. Intracellular ROS production

The ROS generation in MDA-MB-231 cells was measured by DCFH-DA staining. MDA-MB-231 cells were seeded in a 6-well plate at a density of 2×10^5 cells/well and cultured for 12 h at 37 °C. Then, the tested nanoparticles were added with the concentration of 2 μg/mL. After 4 h incubation, DCFH-DA was added and incubated for another 30 min. Thereafter, the cells were washed with fresh medium for three times followed by irradiation with 660 nm laser light at 30 mW/cm^2 for 30 min at a 5 min interval for every 10 min. The stained cells were observed at the wavelength of 488 nm, using a confocal microscope.

14. 3D multicellular tumor spheroids

MDA-MB-231 cancer cells were seeded at a density of 1000 cells/well in round-bottom 96-well plates (Corning Inc., USA) using DMEM media supplemented with

10% FBS at 37 °C to initiate the growth of the spheroids. The growth pattern of the spheroids was monitored for 5 days. At day 5, spheroids were treated with either free Cy5 (5 μM), Cy5-RuCa or Cy5-RuPeg NPs (equal amount of Cy5) under hypoxic conditions for 12 h. After that, Cy5-RuCa and Cy5-RuPeg NPs were irradiated with a 660 nm laser for 10 minutes. After incubating for another 6 h, the amount of Cy5 delivered into the tumor spheroids was visualized by confocal fluorescence microscopy.

15. *In vivo* biodistribution

All animal procedures were performed in accordance with the Guidelines for Care and Use of Laboratory Animals of Southeast University and approved by the Animal Ethics Committee of School of Medicine, Southeast University (Nanjing, China). Mice bearing MDA-MB-231 tumors were intravenously administrated with Cy5-RuPeg or Cy5-RuCa at 5 μg mL^{-1} of Cy5. At the given time intervals, *in vivo* tumor imaging was obtained by IVIS small animal imaging system. After 24 h, mice were sacrificed, major organs (heart, liver, spleen, lung, and kidney) and tumors were collected. The organs and tumors imaging was obtained by IVIS small animal imaging system.

16. *In vivo* antitumor efficacy of CC5-RuCa

The MDA-MB-231 cell xenograft mice model was established as described before. When the tumors reached a volume of 70-100 mm^3 in all mice, animals were randomly divided into six groups, and started on the second day. CC5-RuCa was i.v. injected into mice bearing tumors with a dosage of 15 mg/kg on the first, sixth and eleventh days of the experiment. Then, the tumor sites were irradiated with 660 nm light (0.2 W/cm^2, 10 min) 12 h after injection. Five control experiments were also conducted: (1) vehicle (injected with saline and not irradiated); (2) vehicle+light (injected with saline and irradiation); (3) RuCa (injected with RuCa and not irradiated, 15 mg/kg); (4) RuCa+light (injected with RuCa and irradiation, 15 mg/kg); (5) CC5-RuCa (injected with CC5-RuCa and not irradiated, 15 mg/kg). The tumor volume and body weight were monitored every 3 days. On the 16th day, mice were

sacrificed, and the tumor volume was calculated using the formula (length × width × height/2).

17. Histological analysis

After 16 days of *in vivo* cancer therapy, MDA-MB-231 cell xenograft mice were sacrificed. Tumors were harvested and fixed in 4% paraformaldehyde, followed by paraffin embedding and sectioning into thin slices (10 μm). These paraffin sections were used for immunohistochemistry (IHC) staining using a standard method.[6] The slices were observed using fluorescence microscopy.

Statistical Analysis

Differences among samples were calculated with the two-tailed Student's *t*-test using an independent samples *t*-test in SPSS 16.0. Differences were considered statistically significant at a level of $p < 0.05$ and very significant when $p < 0.01$.

Table S1. IC_{50} values of CI-994, CC4 and CC5 against different cells under normoxia.

Compound	IC_{50} (μM)				
	A549	SGC7901	MCF-7	MDA-MB-231	HUVEC
CC4	>200	>200	>200	>200	>200
CI-994	25.93 ± 2.25	57.77 ± 2.52	43.06 ± 2.28	15.11 ± 1.58	>200
CC5	6.31 ± 0.41	15.48 ± 1.17	11.10 ± 0.89	10.48 ± 0.76	54.95 ± 3.27

Table S2. IC_{50} values of CI-994, CC4 and CC5 against different cells under hypoxia.

Compound	IC_{50} (μM)				
	A549	SGC7901	MCF-7	MDA-MB-231	HUVEC
CC4	>200	>200	>200	>200	>200
CI-994	30.63 ± 1.89	51.34 ± 4.05	50.66 ± 2.78	26.12± 0.95	>200
CC5	4.26 ± 0.61	10.98 ± 1.78	3.17 ± 0.49	0.34 ± 0.11	35.24 ± 2.83

Table S3. Cytotoxicity of RuCa and CC5-RuCa against MDA-MB-231 cells with and without light irradiation under normoxic or hypoxic condition.

Compound	IC_{50} (μg/mL)			
	Normoxia		Hypoxia	
	Dark	660 nm	Dark	660 nm
RuCa	>100	42.37 ± 3.05	>100	68.04 ± 4.92
CC5-RuCa	>100	5.16 ± 0.63	>100	0.81 ± 0.12

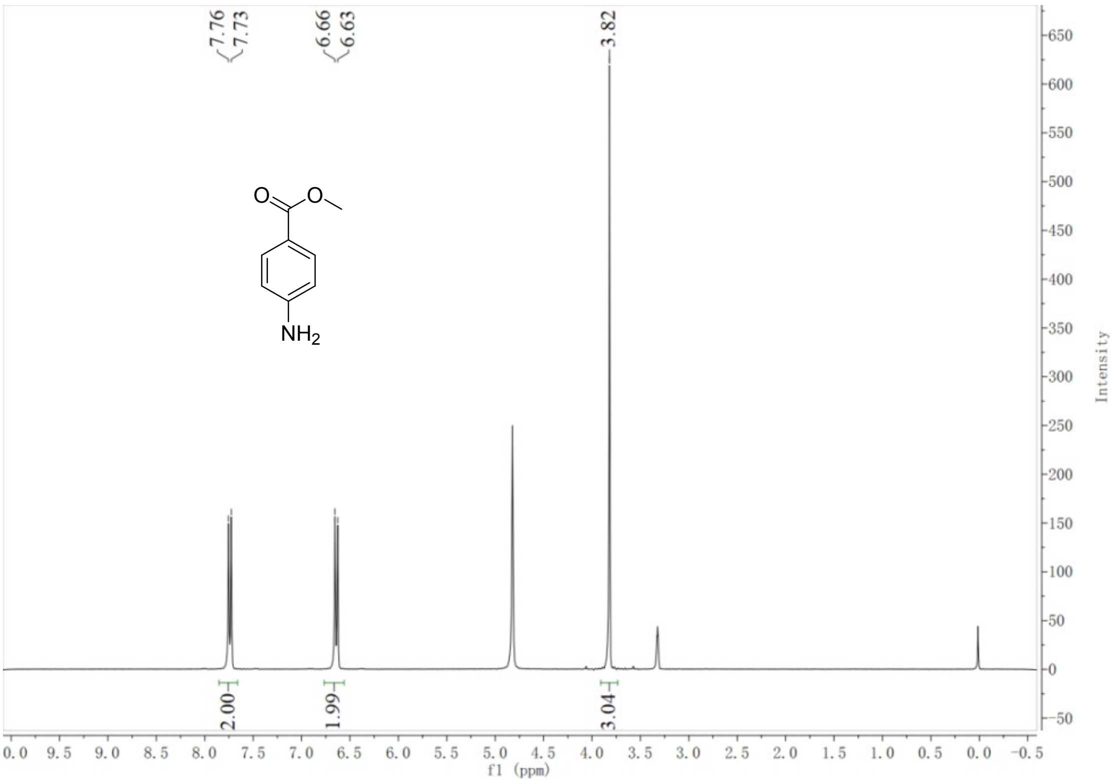

Figure S1. ^{1}H NMR spectrum of CC1 (300 MHz, Methanol-d_4)

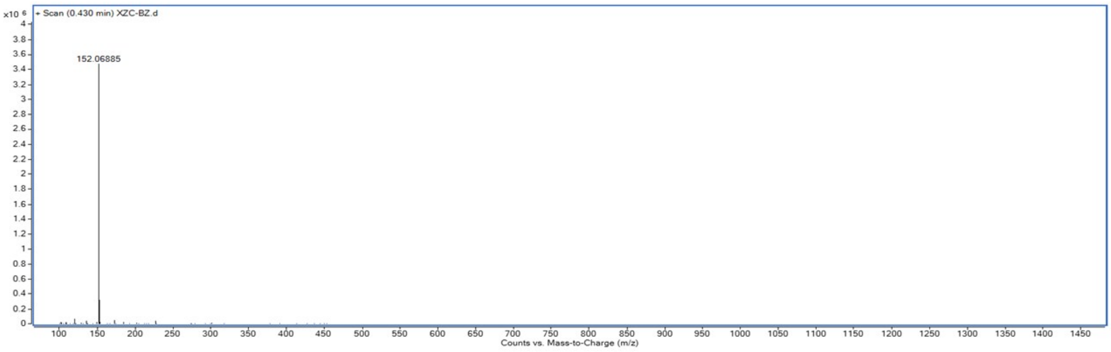

Figure S2. HR-MS mass spectrum of CC1.

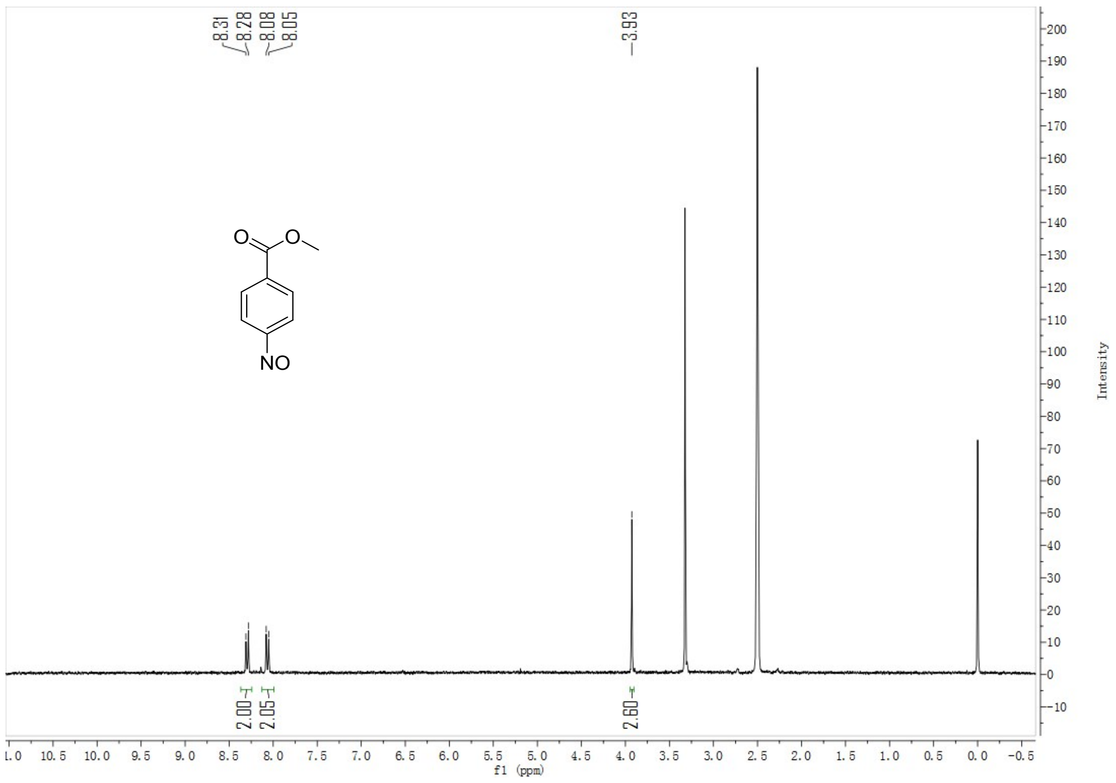

Figure S3. ^{1}H NMR spectrum of CC2 (300 MHz, DMSO-d_6)

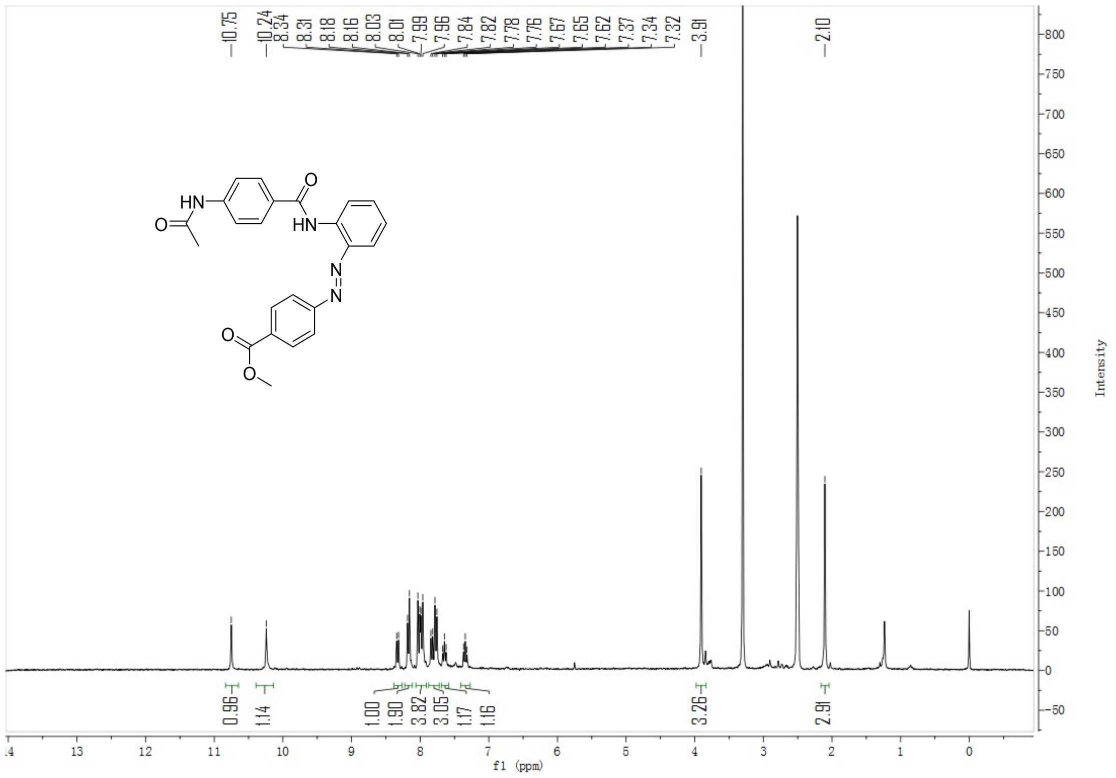

Figure S4. ^{1}H NMR spectrum of CC3 (300 MHz, Methanol-d_4)

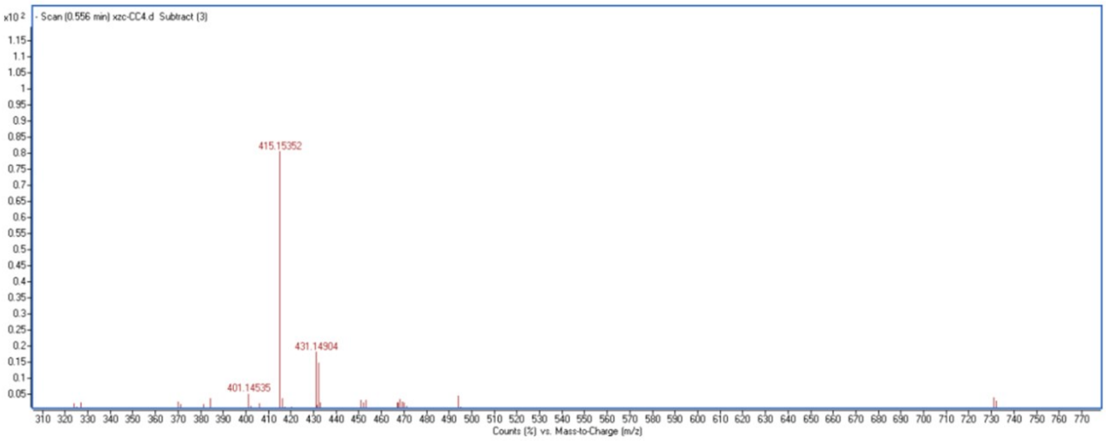

Figure S5. HR-MS mass spectrum of CC3.

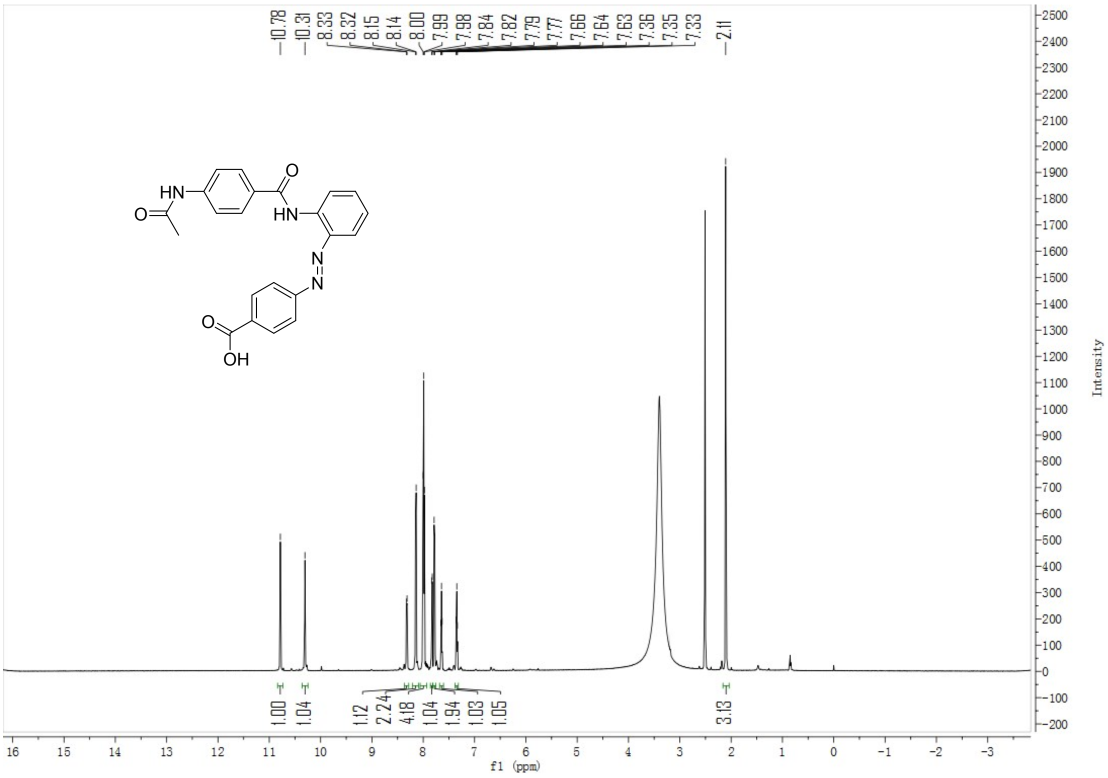

Figure S6. ^{1}H NMR spectrum of CC4 (600 MHz, DMSO-d_6)

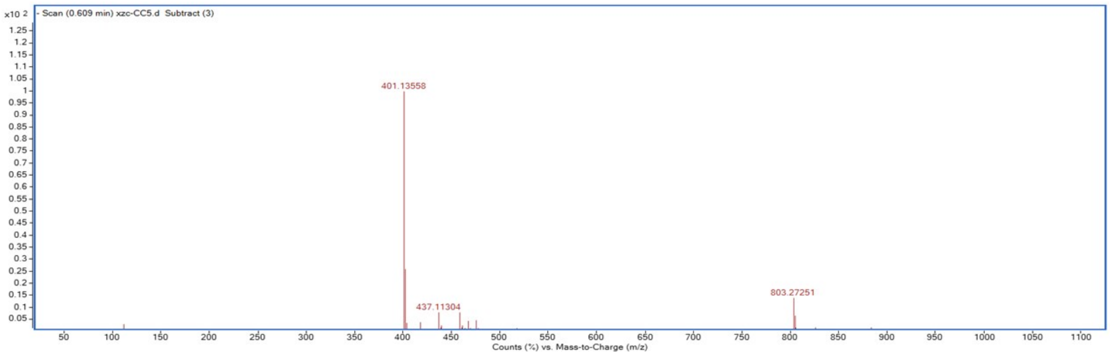

Figure S7. HR-MS mass spectrum of CC4.

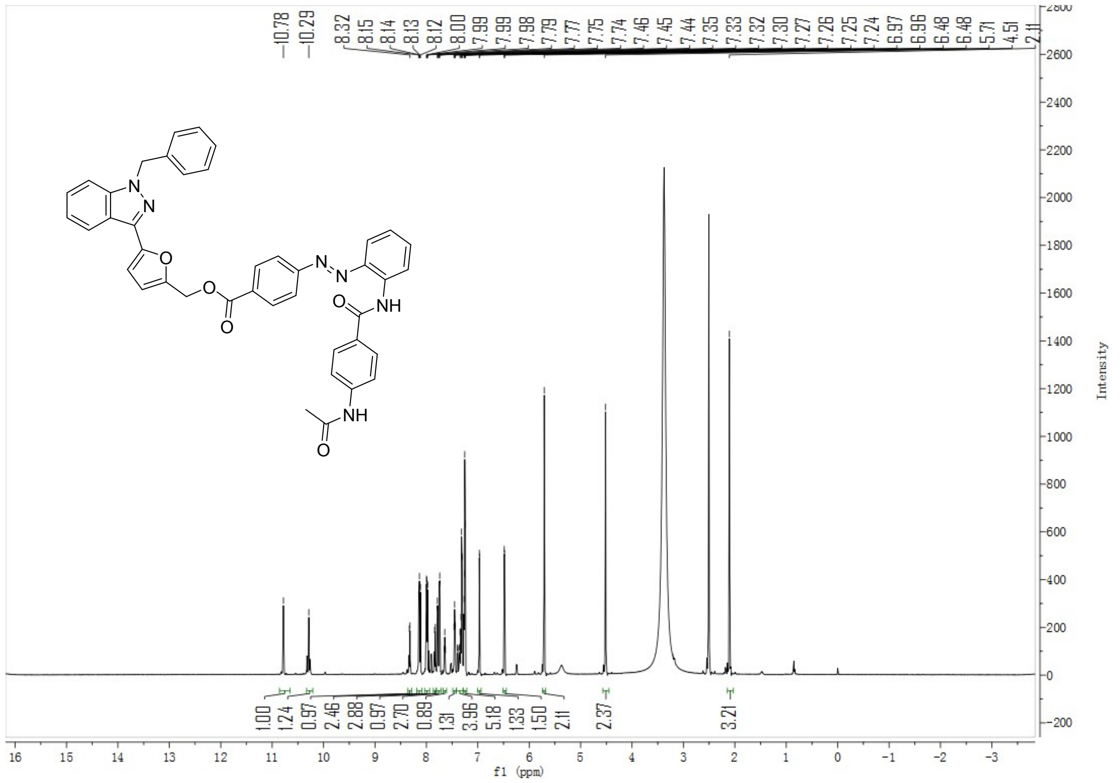

Figure S8. ^{1}H NMR spectrum of CC5 (600 MHz, DMSO-d_6)

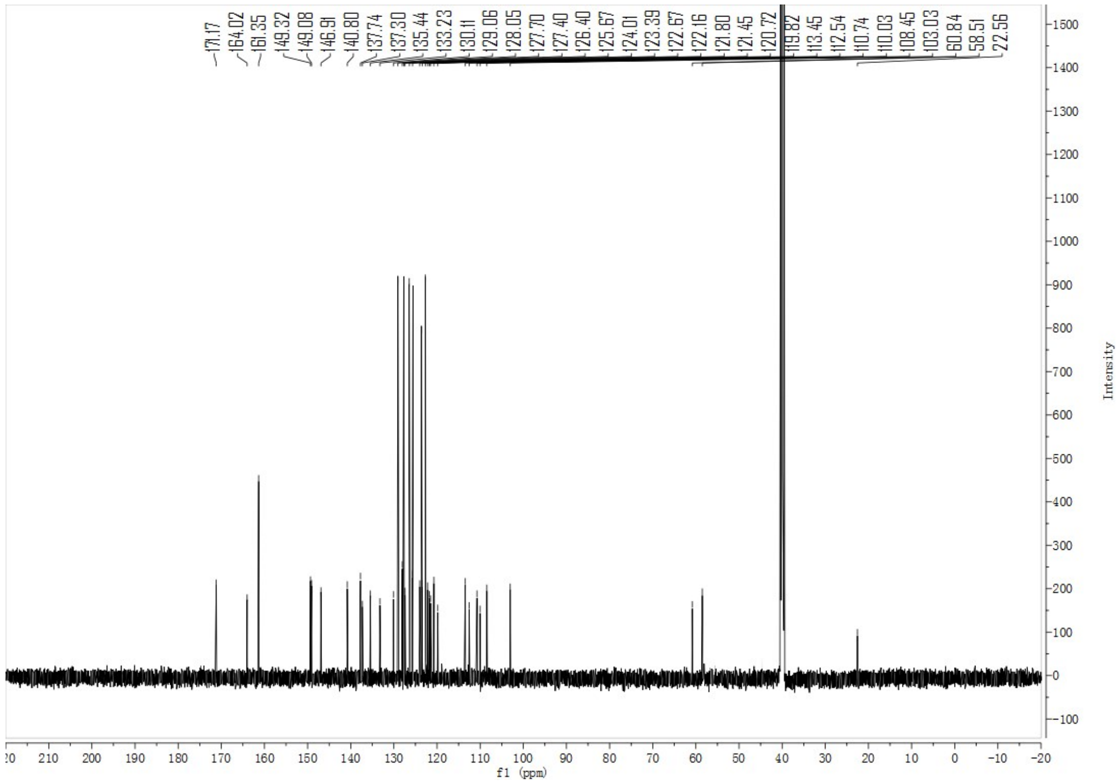

Figure S9. ^{13}C NMR spectrum of CC5 (150 MHz, DMSO-d_6)

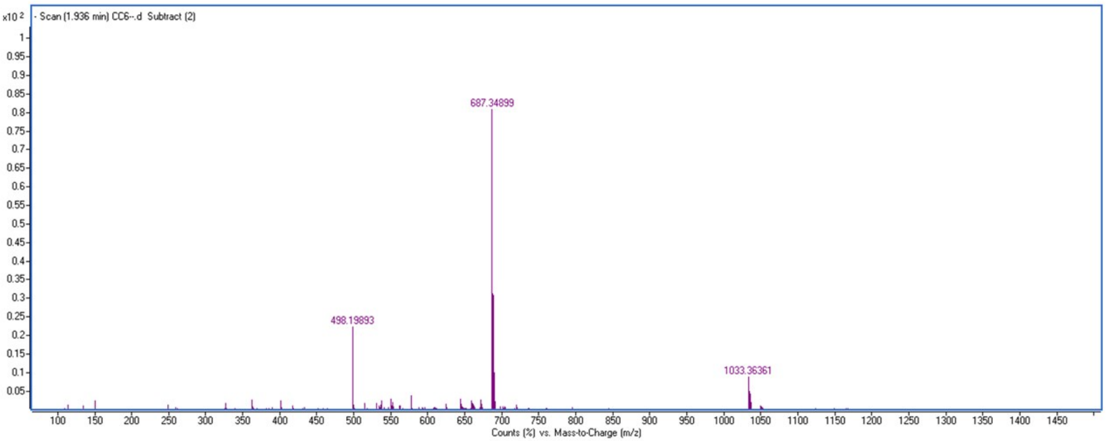

Figure S10. HR-MS mass spectrum of CC5.

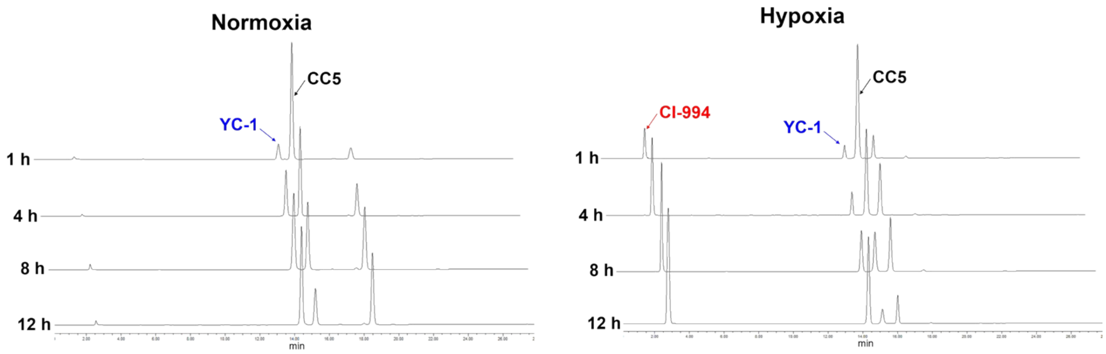

Figure S11. HPLC diagrams of CC5 under normoxic or hypoxic microenvironment at pH 5.0.

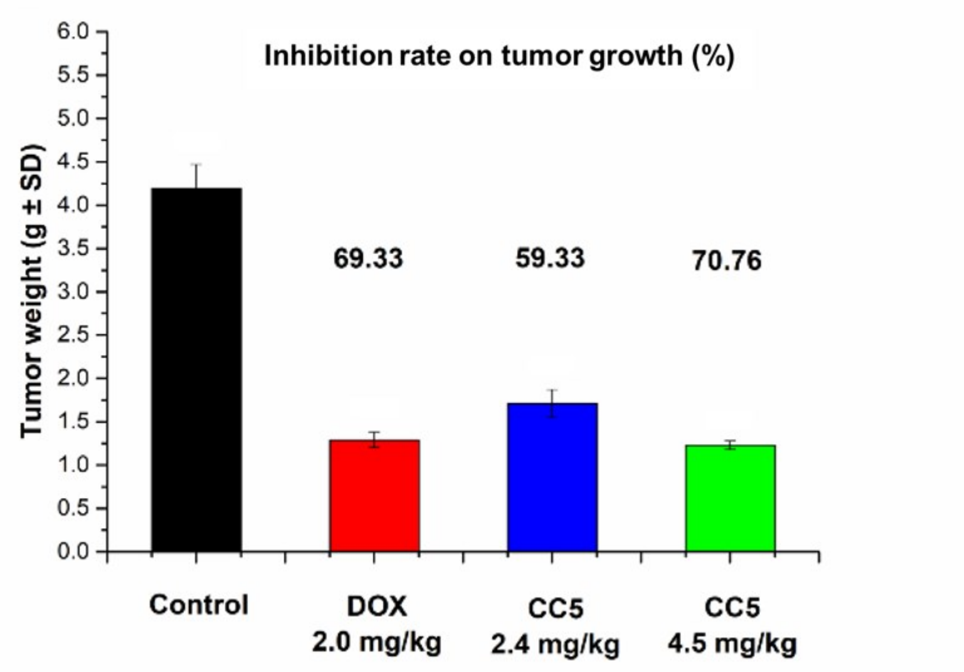

Figure S12. Tumor weight of the mice from each group after treated with CC5 and DOX.

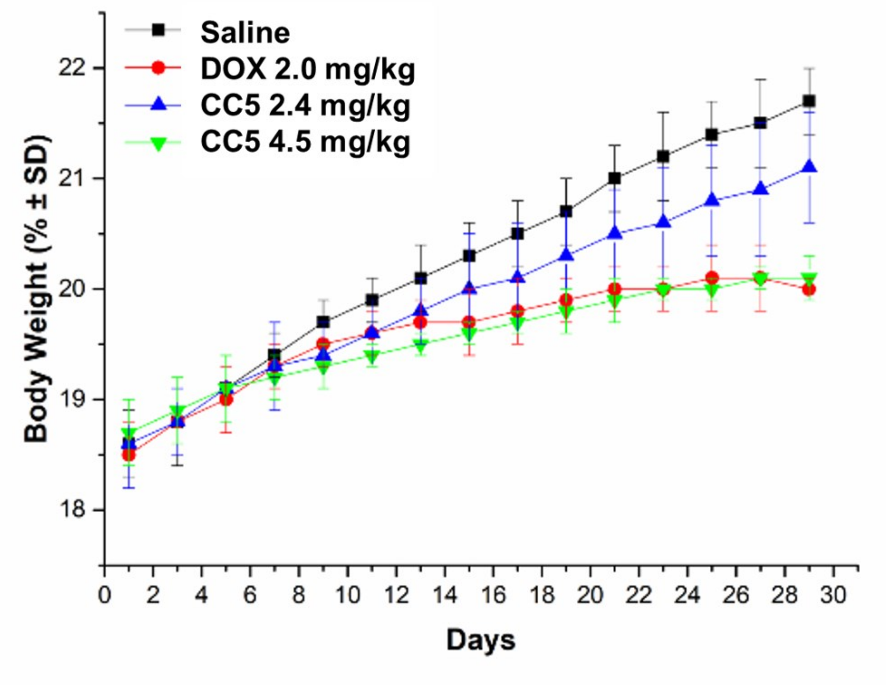

Figure S13. Body weight of the excised mice of each group after treated with CC5 and DOX.

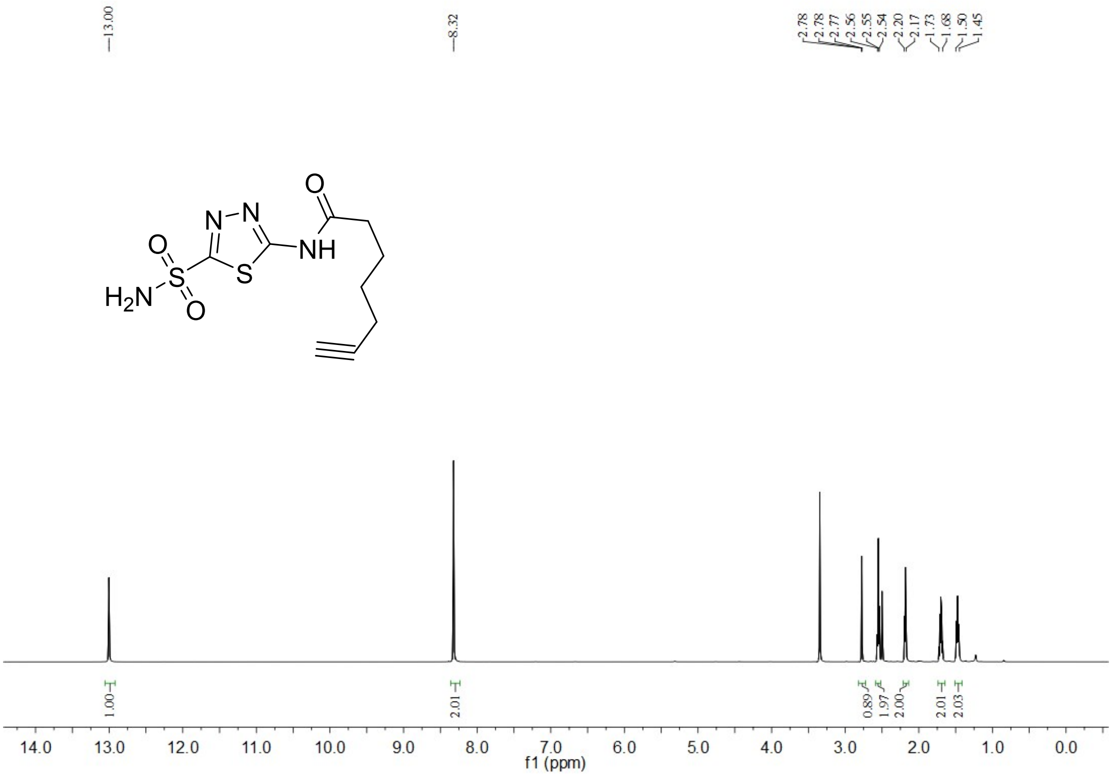

Figure S14. ^{1}H NMR spectrum of CA-2 (600 MHz, DMSO-d_6)

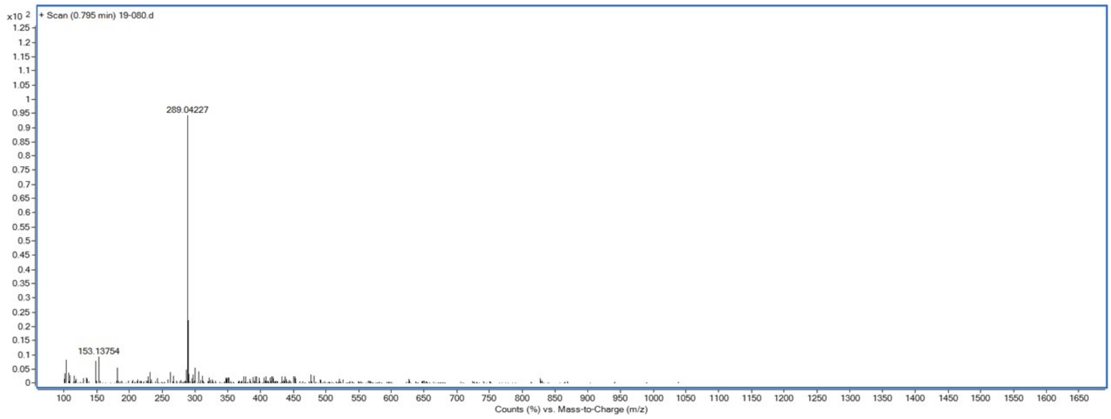

Figure S15. HR-MS mass spectrum of CA-2.

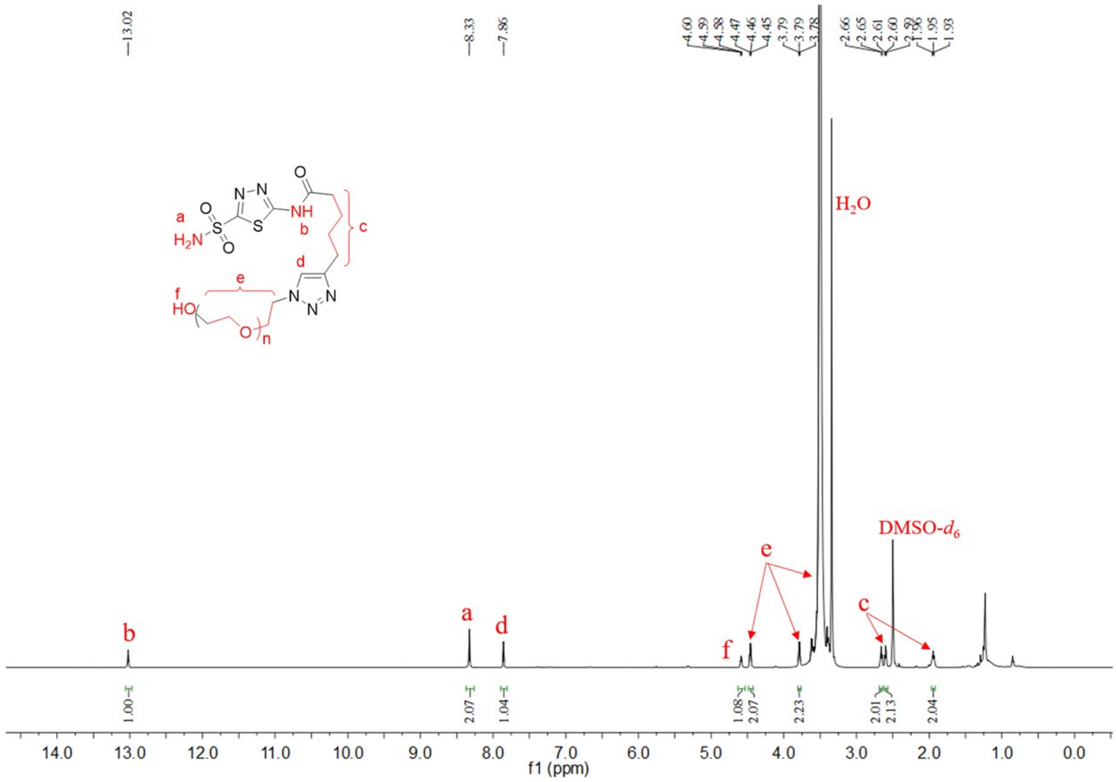

Figure S16. ^{1}H NMR spectrum of CA-PEG (600 MHz, DMSO-d_6)

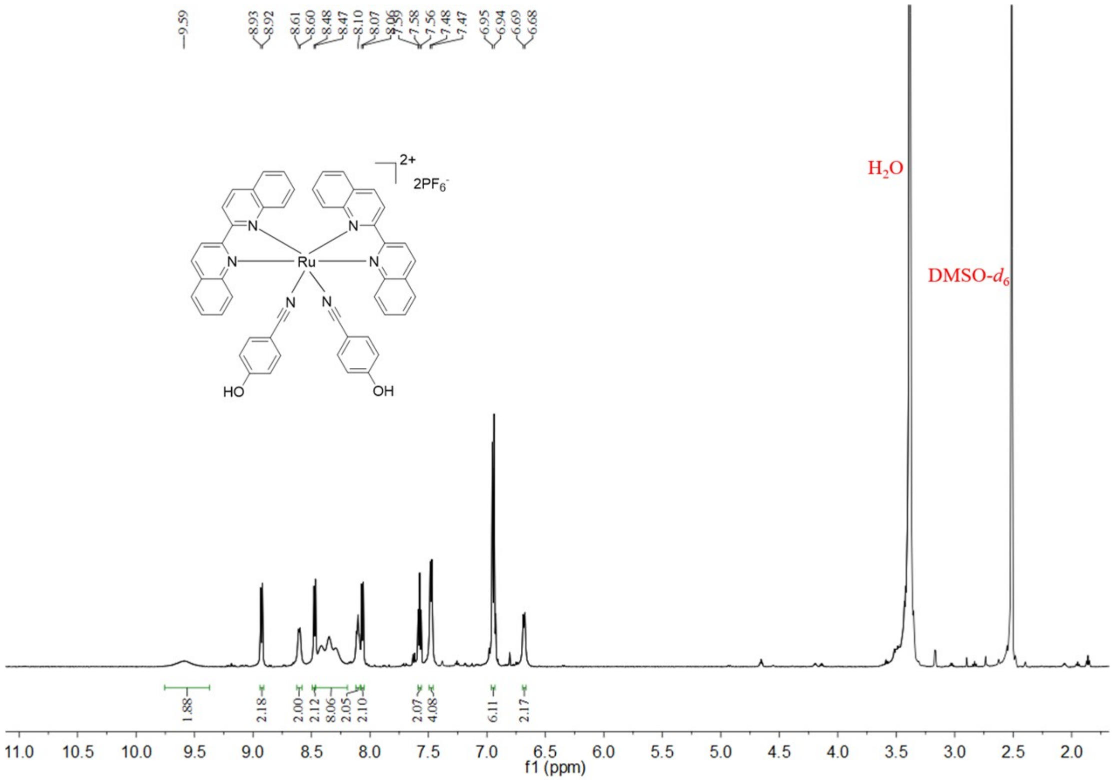

Figure S17. ^{1}H NMR spectrum of $[Ru(Biq)_2(Cya)_2](PF_6)_2$ (600 MHz, DMSO-d_6)

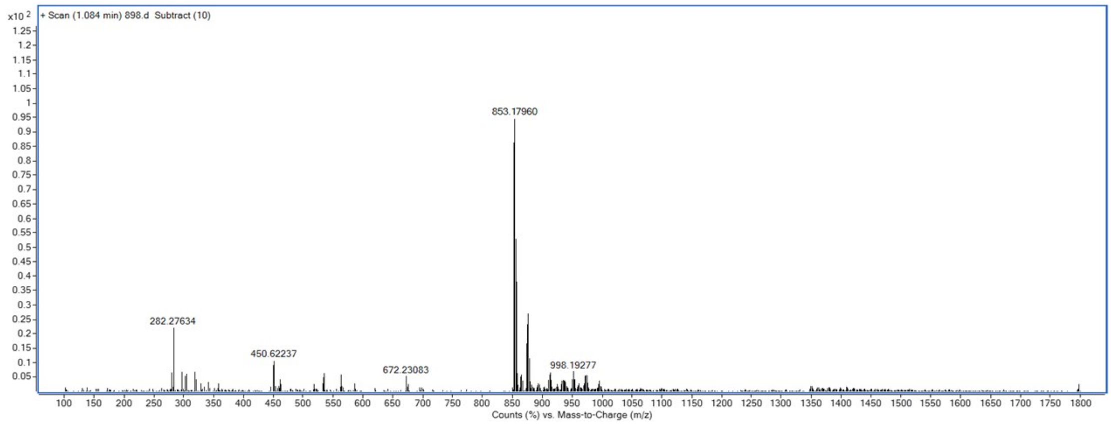

Figure S18. HR-MS mass spectrum of $[Ru(Biq)_2(Cya)_2](PF_6)_2$.

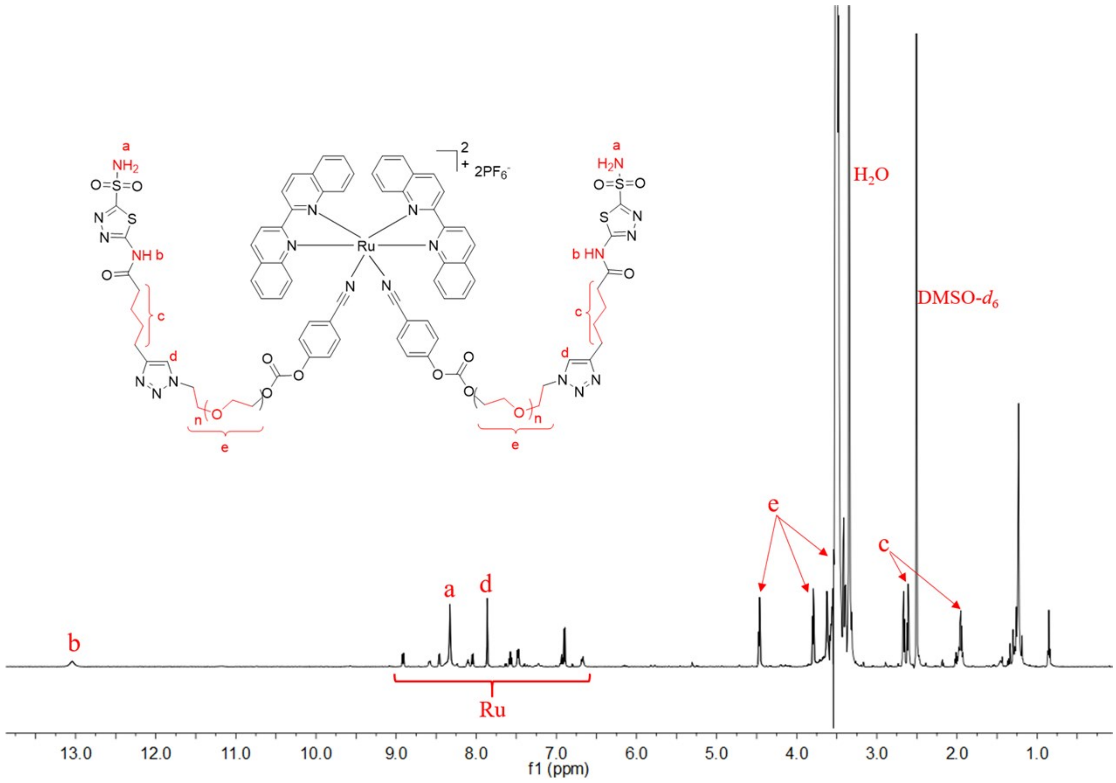

Figure S19. ^{1}H NMR spectrum of RuCa (600 MHz, DMSO-d_6)

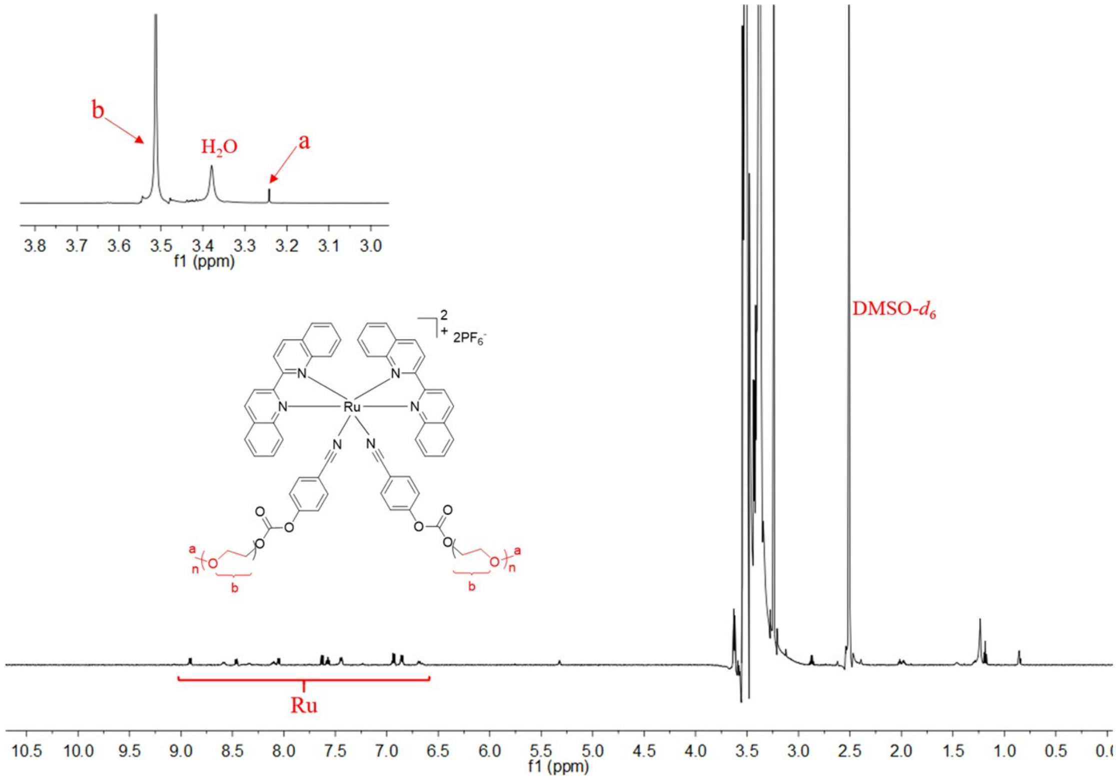

Figure S20. ^{1}H NMR spectrum of RuPeg (600 MHz, DMSO-d_6)

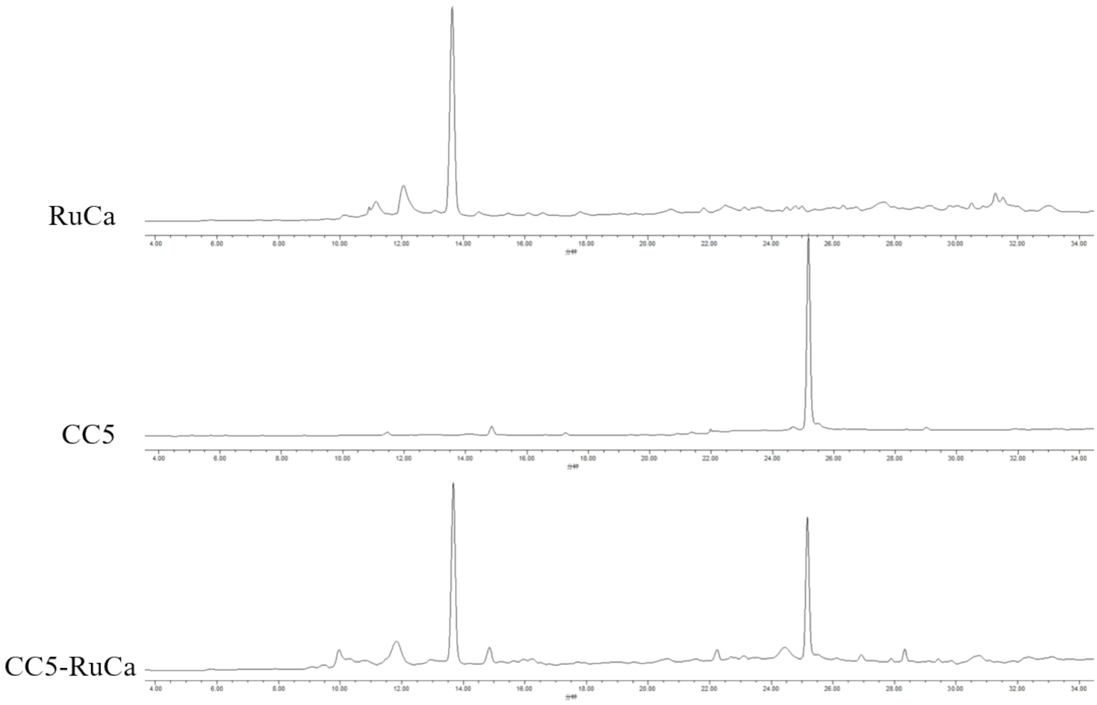

Figure S21. HPLC diagrams of CC5-RuCa (H_2O:MeOH = 90:10 to 0:100).

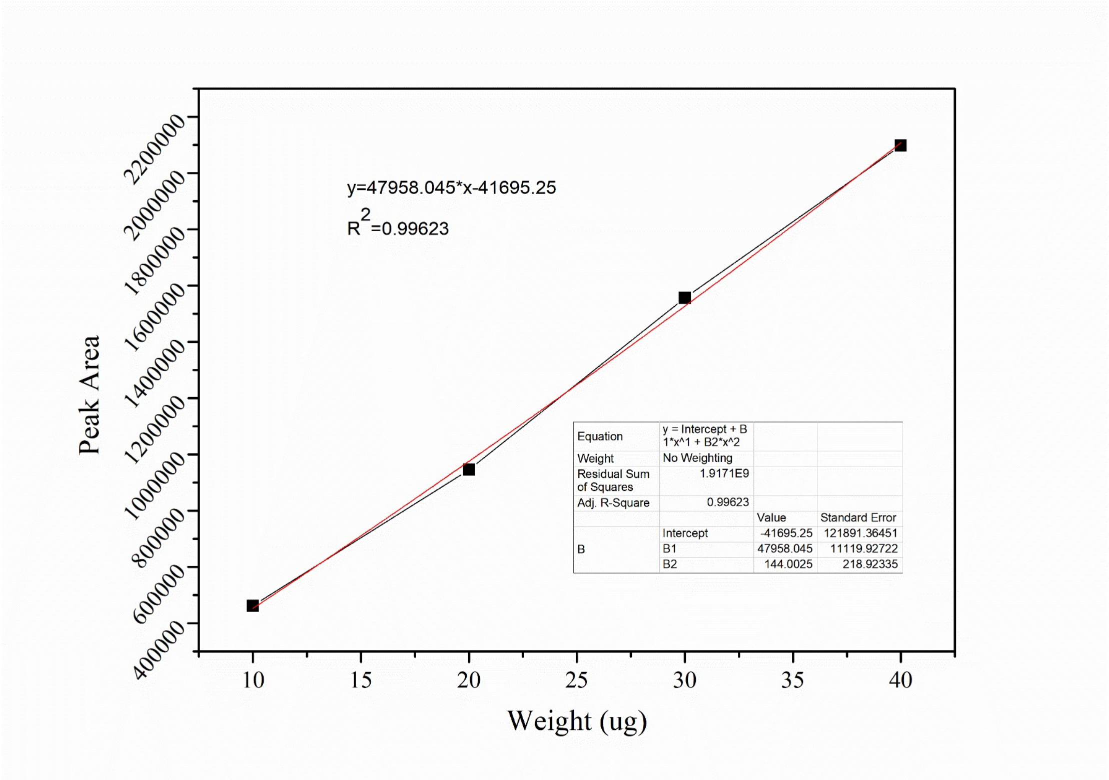

Figure S22. The standard curve of CC5 determined by HPLC with a UV detection at 254 nm.

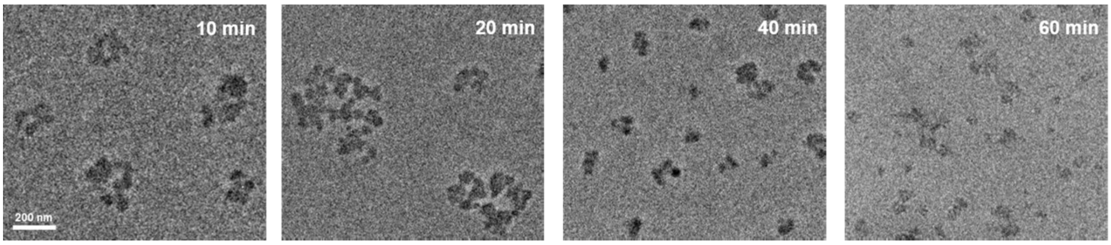

Figure S23. TEM images of RuCa NPs under different irradiation time. Scale bar: 200 nm.

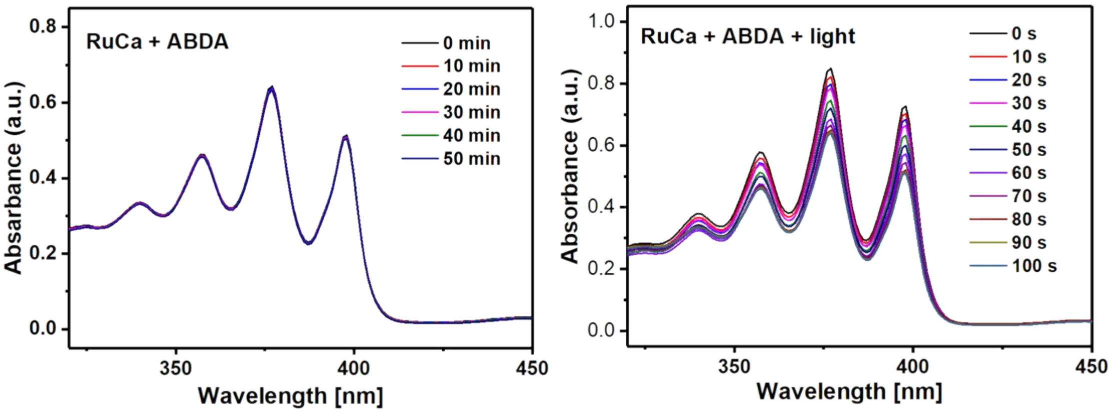

Figure S24. Absorption spectra of ABDA (50 μM) for RuCa (25 μg/mL) with or without 660 nm (30 mW/cm^2) light irradiation.

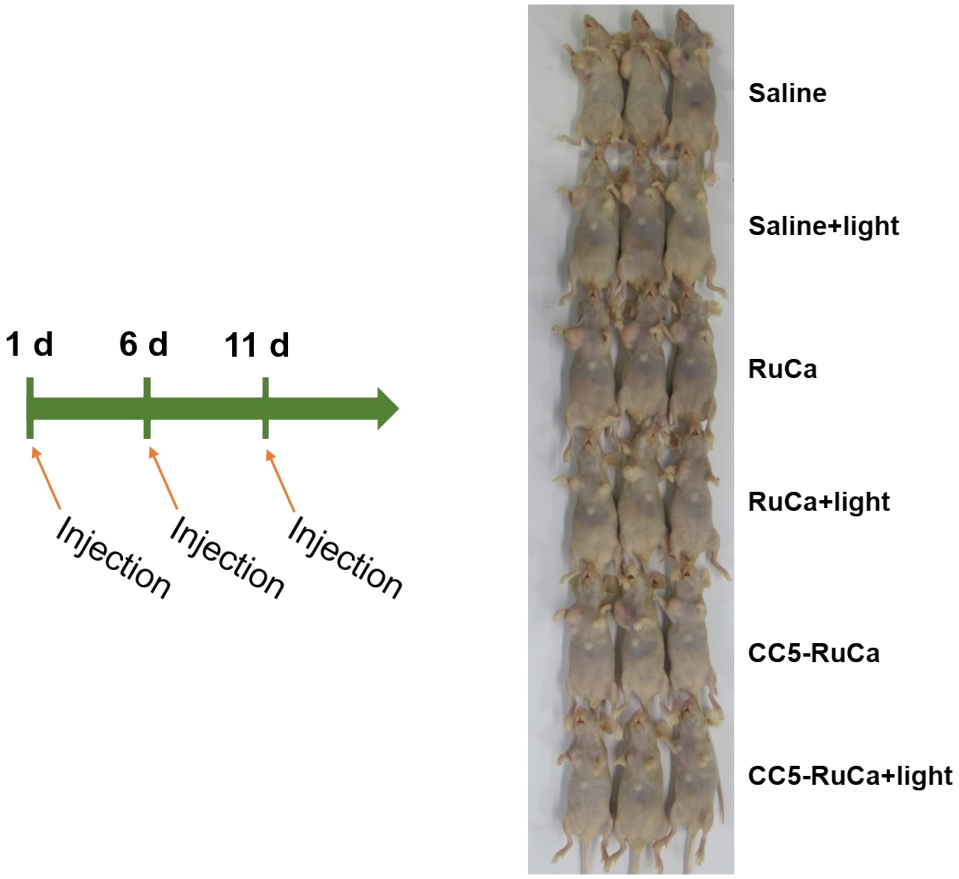

Figure S25. Schematic illustration of drug administration and images of the mice after different treatments for 16 days.

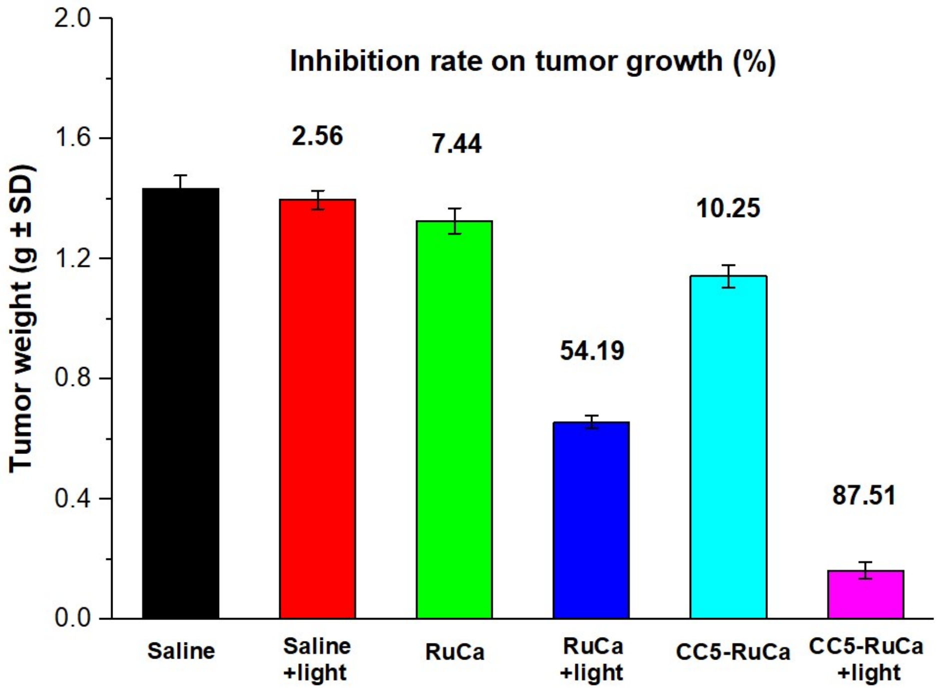

Figure S26. Tumor weight of the mice from each group at the end of the observation period.

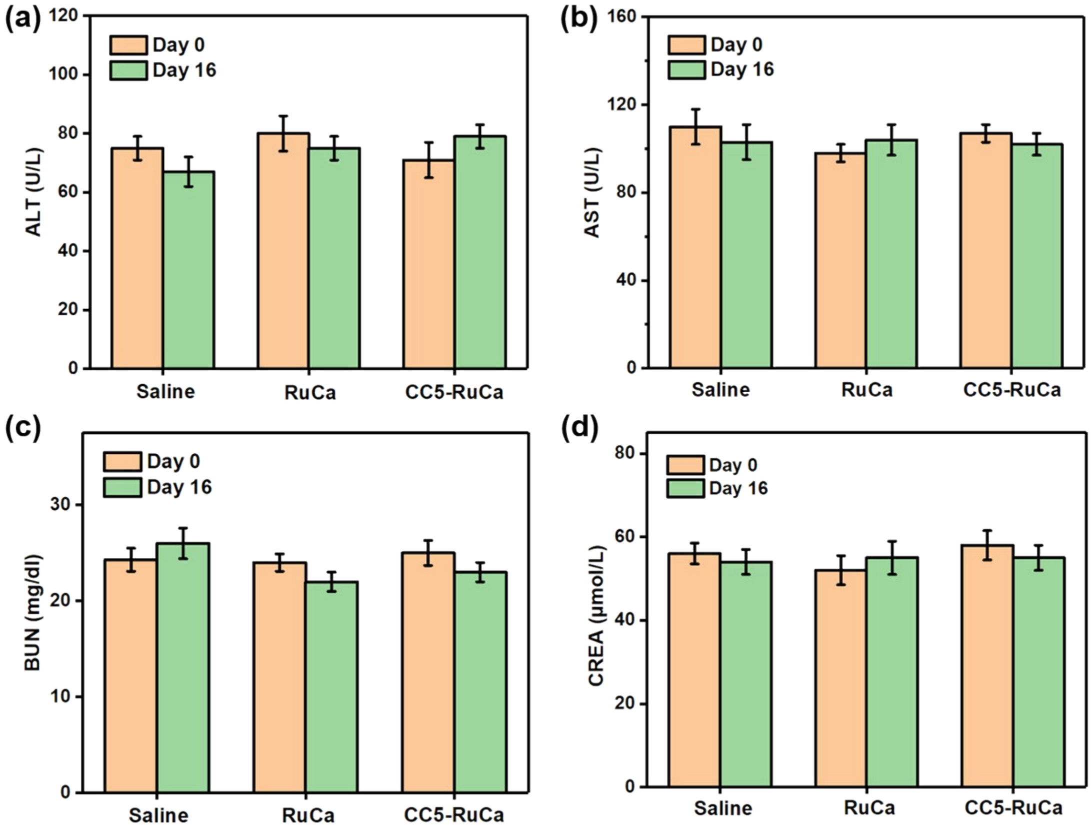

Figure S27. Blood biochemical analysis of mice after adminstration of RuCa or CC5-RuCa NPs. (a) Glutamic-pyruvic transaminase (ALT) and (b) glutamic oxalacetic transaminase (AST) concentrations as indicators for liver function. (c) Urea nitrogen (BUN) and (d) creatinine (CREA) concentrations as indicators for renal function. The data represent the means ± s.d., $n = 3$.

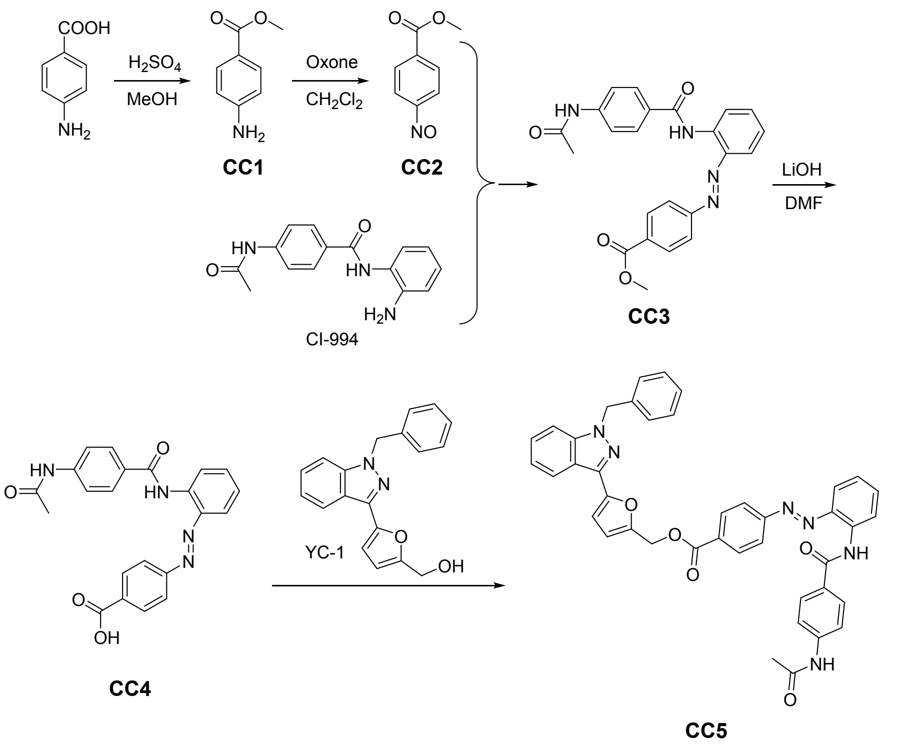

Scheme S1. Preparation of CC5.

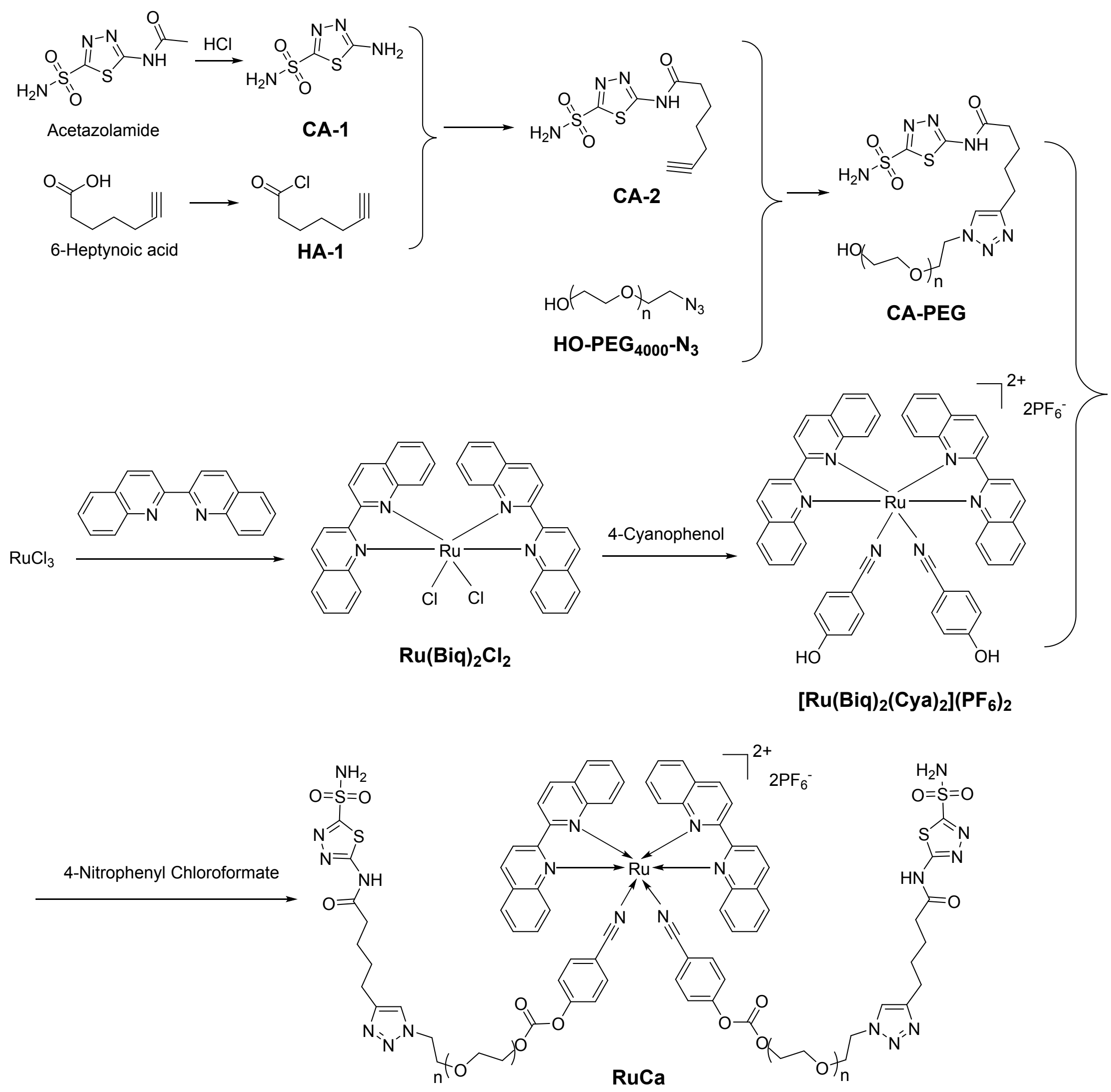

Scheme S2. Preparation of RuCa.

References

1 Z. Xu, J. Zhao, S. Gou and G. Xu, *Chem. Commun.*, 2017, **53**, 3749–3752.

2 B. Zhang, X. Huang, H. Wang and S. Gou, *J. Mater. Chem. B*, 2019, **7**, 5352–5362.

3 M. Thomas, J. Clarhaut, I. Tranoy-Opalinski, J. P. Gesson, J. Roche and S. Papot, *Bioorg. Med. Chem.*, 2008, **16**, 8109–8116.

4 R. P. Tanpure, B. Ren, T. S. Peat, L. F. Bornaghi, D. Vullo, C. T. Supuran and S. A. Poulsen, *J. Med. Chem.*, 2015, **58**, 1494–1501.

5 B. A. Albani, C. B. Durr and C. Turro, *J. Phys. Chem. A*, 2013, **117**, 13885−13892.

6 B. Q. Spring, I. Rizvi, N. Xu and T. Hasan, *Photoch. Photobio. Sci.*, 2015, **14**, 1476−1491.
